# Methods to Establish Race or Ethnicity of Twitter Users: Scoping Review

**DOI:** 10.2196/35788

**Published:** 2022-04-29

**Authors:** Su Golder, Robin Stevens, Karen O'Connor, Richard James, Graciela Gonzalez-Hernandez

**Affiliations:** 1 Department of Health Sciences University of York York United Kingdom; 2 School of Communication and Journalism University of Southern California Los Angeles, CA United States; 3 Department of Biostatistics, Epidemiology, and Informatics, Perelman School of Medicine University of Pennsylvania Philadelphia, PA United States; 4 School of Nursing Liaison and Clinical Outreach Coordinator University of Pennsylvania Philadelphia, PA United States

**Keywords:** twitter, social media, race, ethnicity

## Abstract

**Background:**

A growing amount of health research uses social media data. Those critical of social media research often cite that it may be unrepresentative of the population; however, the suitability of social media data in digital epidemiology is more nuanced. Identifying the demographics of social media users can help establish representativeness.

**Objective:**

This study aims to identify the different approaches or combination of approaches to extract race or ethnicity from social media and report on the challenges of using these methods.

**Methods:**

We present a scoping review to identify methods used to extract the race or ethnicity of Twitter users from Twitter data sets. We searched 17 electronic databases from the date of inception to May 15, 2021, and carried out reference checking and hand searching to identify relevant studies. Sifting of each record was performed independently by at least two researchers, with any disagreement discussed. Studies were required to extract the race or ethnicity of Twitter users using either manual or computational methods or a combination of both.

**Results:**

Of the 1249 records sifted, we identified 67 (5.36%) that met our inclusion criteria. Most studies (51/67, 76%) have focused on US-based users and English language tweets (52/67, 78%). A range of data was used, including Twitter profile metadata, such as names, pictures, information from bios (including self-declarations), or location or content of the tweets. A range of methodologies was used, including manual inference, linkage to census data, commercial software, language or dialect recognition, or machine learning or natural language processing. However, not all studies have evaluated these methods. Those that evaluated these methods found accuracy to vary from 45% to 93% with significantly lower accuracy in identifying categories of people of color. The inference of race or ethnicity raises important ethical questions, which can be exacerbated by the data and methods used. The comparative accuracies of the different methods are also largely unknown.

**Conclusions:**

There is no standard accepted approach or current guidelines for extracting or inferring the race or ethnicity of Twitter users. Social media researchers must carefully interpret race or ethnicity and not overpromise what can be achieved, as even manual screening is a subjective, imperfect method. Future research should establish the accuracy of methods to inform evidence-based best practice guidelines for social media researchers and be guided by concerns of equity and social justice.

## Introduction

### Research Using Twitter Data

Twitter data are increasingly being used as a surveillance and data collection tool in health research. When millions of users post on Twitter, it translates into a vast amount of publicly accessible, timely data about a variety of attitudes, behaviors, and preferences in a given population. Although these data were not originally intended as a repository of individual information, Twitter data have been retrofitted in infodemiology to investigate population-level health trends [1-15]. Researchers often use Twitter data in consort with other sources to test the relationship between web-based discourse and offline health behavior, public opinion, and disease incidence.

The appeal of Twitter data is clear. Twitter is one of the largest public-facing social media platforms, with an ethnically diverse user base [16,17] of more than 68 million US Twitter users, with Black users accounting for 26% of that base [18]. This diverse user base gives researchers access to people they may have difficulty reaching using more traditional approaches [19]. However, promising insights that can be derived from Twitter data are often limited by what is missing, specifically the basic sociodemographic information of each Twitter user. The demographic attributes of users are often required in health research for subpopulation analyses, to explore differences, and to identify inequity. Without evidence of the distal and proximal factors that lead to racial and ethnic health disparities, it is impossible to address and correct these drivers. Insights from social media data can be used to inform service provision as well as to develop targeted health messaging by understanding public perspectives from diverse populations.

### Extracting Demographics From Twitter

However, to use social media and digital health research to address disparities, we need to know not only what is said on Twitter but also who is saying what [20]. Although others have discussed extracting or estimating features, such as location, age, gender, language, occupation, and class, no comprehensive review of the methods used to extract race or ethnicity has been conducted [20]. Extracting the race and ethnicity of Twitter users is particularly important for identifying trends, experiences, and attitudes of racially and ethnically diverse populations [21]. As race is a social construction and not a genetic categorization [22,23], the practice of defining race and ethnicity in health research has been an ongoing, evolving challenge. Traditional research has the advantage of identifying the person in the study and allowing them to systematically identify their racial and ethnic identities. In digital health research [22,23], determining a user’s race or ethnicity by extracting data from a user’s Twitter profile, metadata, or tweets is a process that is inevitably challenging, complex, and not without ethical questions.

Furthermore, although Twitter is used for international research, an international comparative study of methods to determine race or ethnicity is difficult, practically impossible, given that societies use different standardized categories that describe their own populations [24]. A common approach in the United States is based on the US Census Bureau practice to allow participants to identify with as many as 5-6 large racial groupings (Black, White, Asian, Pacific Islander, Native, and other), while separately choosing one ethnicity (Hispanic) [25]. However, race and ethnicity variables continue to be misused in the study design or when drawing conclusions. For example, race or ethnicity is often incorrectly treated as a predictor of poor health rather than as a proxy for the impact of being a particular race or ethnicity has on that person’s experience with the health system [26]. Simply put, health disparities are driven by racism, not race [27-29]. Although race or ethnicity affiliation is an important factor in understanding diverse populations, digital research must tread lightly and thoughtfully both the collection and assignment of race or ethnicity.

### Objectives

The lack of basic sociodemographic data on Twitter users has led researchers to apply a variety of approaches to better understand the characteristics of the people behind each tweet. The breadth of the landscape of approaches to extracting race or ethnicity is currently unknown. Our overall aim was to summarize and assess the range of computational and manual methods used in research based on Twitter data to determine the race or ethnicity of Twitter users.

## Methods

### Overview

We conducted a comprehensive scoping review of extraction methods and offered recommendations and cautions related to these approaches [30]. We selected Twitter, as it is currently the most commonly used social media platform in health care research, and it has some unique intrinsic characteristics that drive the methods used for mining it. Thus, we felt that the methods, type of data, and social media platforms used are related in such a way that comparing methods for different social media would add too many variables and would not be truly comparing like with like. A detailed protocol was designed for the methods to be used in our scoping review, but we were unable to register scoping reviews on PROSPERO. We report our methods according to the PRISMA (Preferred Reporting Items for Systematic Reviews and Meta-Analyses) scoping review statement [30].

### Inclusion Criteria

#### Overview

We devised strict inclusion criteria for our review based on the Population, Intervention, Comparators, Outcomes, and Study design format. Although this was not a review of effectiveness, we felt that the Population, Intervention, Comparators, Outcomes, and Study design question breakdown [31] was still the most appropriate one available for our question format [31]. The inclusion criteria are described in the following sections.

#### Population

We included only data sets of Twitter users. Studies were eligible for inclusion if they collected information to extract or infer race or ethnicity directly from the users’ tweets, their profile details (such as the users’ photo or avatar, their name, location, and biography [bio]), or their followers. We excluded studies that extracted race or ethnicity from social media platforms other than Twitter, from unspecified social media platforms, or those that used multiple social media platforms that included Twitter, but the data relating to Twitter were not presented separately.

#### Intervention

Studies were included where the methods to extract or infer the race or ethnicity data of Twitter users were stated. Articles that used machine learning (ML), natural language processing (NLP), human-in-the-loop, or other computationally assisted methods to predict race or ethnicity of users were included, as were manual or noncomputational methods, including photo recognition or linking to census data. We excluded studies for which we were unable to determine the methods used or for which we extracted data solely on other demographic characteristics, such as age, gender, or geographic location.

#### Comparator

The use of a comparison of the methods used was not required. A method could be compared with another (such as a gold standard), or no comparison could be undertaken.

#### Outcome

The extraction or inference of the race or ethnicity of Twitter users was the primary or secondary outcome of the study. As this was a scoping review in which we aimed to demonstrate the full landscape of the literature, no particular measurement of the performance of the method used was required in our included studies.

#### Study Design

Any type of research study design was considered relevant. Discussion papers, commentaries, and letters were excluded.

### Limits

No restrictions on date, language, or publication type were applied to the inclusion criteria. However, no potentially relevant studies were identified in any non-English language, and the period by default was since 2006, the year of the inception of Twitter.

### Search Strategy

A database search strategy was derived by combining three facets: facet 1 consisted of free-text terms related to Twitter (*Twitter* OR Tweet* OR *Tweeting* OR Retweet* OR Tweep*); facet 2 consisted of terms for race or ethnicity; and facet 3 consisted of terms for methods of prediction, such as ML, NLP, and artificial intelligence–related terms (Table S1 in Multimedia Appendix 1 [3,10,12,18,20,21,32-96]). All ethnology-related subject terms were adapted for different database taxonomies and syntax, with standard methods for predicting subject terms in MEDLINE and other database indexing. The methods of predicting term facets were expanded using a comprehensive list of specific text analysis tools and software names extracted from the study by Hinds and Joinson [97], which included a comprehensive list of automated ML processes used in predicting demographic markers in social media. Additional terms have been added from a related study [98].

### Sources Searched

A wide range of bibliographic and gray literature databases were selected to search for topics on computer science, health, and social sciences. The databases (Table 1) were last searched on May 15, 2021, with no date or other filter applied.

Reference checking of all included studies and any related systematic reviews identified by the searches were conducted. We browsed the Journal of Medical Internet Research, as this is a key journal in this field, and hand searched 2 relevant conferences, the International Conference on Weblogs and Social Media and Association for Computational Linguistics proceedings.

Citations were exported to a shared Endnote library, and duplicates were removed. The deduplicated records were then imported into Rayyan to facilitate independent blinded screening by the authors. Using the inclusion criteria, at least two screeners (SG, RS, KO, or RJ) from the research team independently screened each record, with disputes on inclusion discussed and a consensus decision reached.

Only the first 50 records from ACL and the first 100 records from a Google Scholar search were screened during two searches (March 11, 2020, and May 24, 2021) as these records are displayed in order of relevance, and it was felt that after this number no relevant studies were being identified [12,21,32-95,99].

**Table 1:** Databases searched with number of records retrieved.

Database	Total results, n
ACL Anthology	Screened first 50 records from 2 searches
ACM Digital Library	150
CINAHL	200
Conference Proceedings Citation Index—Science	84
Conference Proceedings Citation Index—Social Science	7
Emerging Sources Citation Index	41
Google Scholar	Screened first 100 records from 2 searches
IEEE Xplore	186
Library and Information Science Abstracts	120
LISTA	79
OpenGrey	0
ProQuest dissertations and theses—United Kingdom and Ireland	195
PsycINFO	72
PubMed	84
Science Citation Index	56
Social Science Citation Index	111
Zetoc	50

### Data Extraction

For each included study, we extracted the following data on an excel spreadsheet:

year of publication, study country and language, race or ethnicity categories extracted (such as for race—Black, White, or Asian or for ethnicity—Hispanic or European), and paper type (journal, conference, or thesis). We also extracted details on extraction methods (such as classification models or software used), features and predictors used in extraction (tweets, profiles, and pictures), number of Twitter users, number of tweets or images used, performance measures to evaluate methods used (validation), and results of any evaluation (such as accuracy). All performance measure metrics were reported as stated in the included studies. All the extracted data were checked by 2 reviewers.

### Quality Assessment

There was no formally approved quality assessment tool for this type of study. As this was a scoping review, we did not carry out any formal assessment. However, we assessed any validation performed and whether the methods were reproducible.

### Data Analysis

We have summarized the stated performance of the papers that included validation. However, we could not compare approaches using the stated performance, as the performance measures and validation approaches varied considerably. In addition, there is no recognized gold standard data set for comparison.

## Results

### Overview

**Figure 1:**
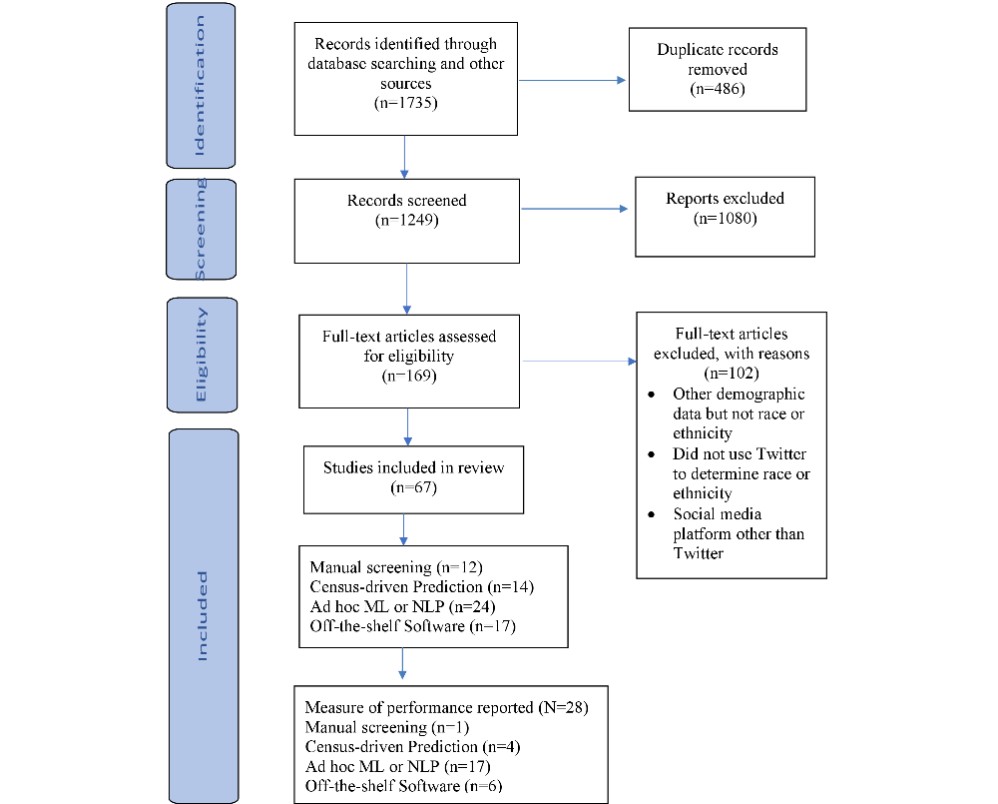
Flow diagram for included studies.

A total of 1735 records were entered into an Endnote library (Clarivate), and duplicates were removed, leaving 1249 (72%) records for sifting (Figure 1). A total of 1080 records were excluded based on the title and abstract screening alone. A total of 169 references were deemed potentially relevant by one of the independent sifters (RS, GG, RJ, SG, and KO). The full text of these articles was screened independently, and 67 studies [12,21,32-95,99] met our inclusion criteria and 102 references were excluded [77,97,100-198]. The main reason for exclusion was that although the abstract indicated that demographic data were collected, it did not include race or ethnicity (most commonly, other demographic attributes such as gender, age, or location were collected). Other reasons for exclusion were that the researchers collected demographic data through surveys or questionnaires administered via Twitter (but not from data posted on Twitter) or that the researchers used a social media platform other than Twitter.

### Characteristics of the Included Studies

Most of the studies (51/67, 76%) stated or implied that they were based solely or predominantly in the United States and were limited to English language bios or tweets. A total of 6 studies were multinational [38,41,56,66,83,86]; 1 was UK based (also in English) [59], another was based in Qatar [55], and 12% (8/67) of studies extracted data from tweets in multiple languages [32,38,52,55,56,66,83,86] (Table S2 in Multimedia Appendix 1).

The most common race examined was White (58/67, 87%), followed by Black or African American (56/67, 84%), Asian (45/67, 67%), and the most common ethnicity examined was Hispanic/Latino (43/67, 64%).

Some studies (12/67, 18%) treated race as a binary classification, such as African American or not or African American or White, whereas others created a multiclass classifier of 3 (15/67, 22%) or 4 classes (33/67, 49%) or a combination of classes. A total of 6 studies identified >4 classes; however, these often included ethnicity or nationality classifiers as well as race [38,48,54,66, 83,95]. Wang and Chi [77] was a conference paper which did not report the race types extracted.

The data objects from Twitter used to extract race or ethnicity varied, with the use of profile pictures or Twitter users’ names being the most common. Others have also used tweets in the users’ timeline, information from Twitter bios, or Twitter users’ locations. Most studies (39/67, 58%) used more than one data object from Twitter data. In addition, the data sets within the studies varied in size between 392 and 168,000,000, with those using manual methods having smaller data sets ranging from just 392 [50] to 4900 [65].

Unfortunately, although performance has been measured in 67% (45/67) of studies (this was inconsistently measured Table 2). The metrics used to report results were particularly varied for studies using ML or NLP and included the *F*_1_ score (which combines precision and recall), accuracy, area under the curve, or mean average precision. Table 2 lists the methods, features, and reported performance of the top model from each study.

**Table 2:** Top system performance within studies using machine learning or natural language processing (result metrics are reflected here as reported in the original publications).

Study	Classifier	ML^a^ model	Features	Results reported		
				Accuracy	*F*_1_ score	Area under curve
Pennacchiotti and Popescu, 2011 [68]	Binary	GBDT^b^	Images, text, topics, and sentiment	N/A^c^	0.66	N/A
Pennacchiotti and Popescu, 2011 [67]	Binary	GBDT	Images, text, topics, sentiment, and network	N/A	0.70	N/A
Bergsma et al, 2013 [38]	Binary	SVM^d^	Names and name clusters	0.85	N/A	N/A
Ardehaly and Culotta, 2017 [35]	Binary	DLLP^e^	Text and images	N/A	0.95 (image); 0.92 (text)	N/A
Volkova and Backrach, 2018 [76]	Binary	LR^f^	Text, sentiment, and emotion	N/A	N/A	0.97
Wood-Doughtry et al, 2018 [79]	Binary	CNN^g^	Name	0.73	0.72	N/A
Saravanan, 2017 [72]	Ternary	CNN	Text	NR^h^	NR	NR
Ardehaly and Culotta, 2017 [33]	Ternary	DLLP	Text and images	N/A	0.84 (image); 0.83 (text)	N/A
Gunarathne et al, 2019 [94]	Ternary	CNN	Text	N/A	0.88	N/A
Wood-Doughtry et al, 2018 [79]	Ternary	CNN	Name	0.62	0.43	N/A
Culotta et al, 2016 [47]	Quaternary	Regression	Network and text	N/A	0.86	N/A
Chen et al, 2015 [46]	Quaternary	SVM	n-grams, topics, self-declarations, and image	0.79	0.79	0.72
Markson, 2017 [61]	Quaternary	CNN	Synonym expansion and topics	0.76	N/A	N/A
Wang et al, 2016 [189]	Quaternary	CNN	Images	0.84	N/A	N/A
Xu et al, 2016 [82]	Quaternary	SVM	Synonym expansion and topics	0.76	N/A	N/A
Ardehaly and Culotta, 2015 [34]	Quaternary	Multinomial logistic regression	Census, name, network, and tweet language	0.83	N/A	N/A
Ardehaly, 2014 [64]	Quaternary	LR	Census and image tweets	0.82	0.81	N/A
Barbera, 2016 [37]	Quaternary	LR with EN^i^	Tweets, emojis, and network	0.81	N/A	N/A
Wood-Doughty 2020 [81]	Quaternary	CNN	Name, profile metadata, and text	0.83	0.46	N/A
Preotiuc-Pietro and Ungar, 2018 [96]	Quaternary	LR with EN	Text, topics, sentiment, part-of-speech tagging, name, perceived race labels, and ensemble	N/A	N/A	0.88 (African American), 0.78 (Latino), 0.83 (Asian), and 0.83 (White)
Mueller et al, 2021 [91]	Quaternary	CNN	Text and accounts followed	N/A	0.25 (Asian), 0.63 (African American or Black), 0.28 (Hispanic), and 0.90 (White)	N/A
Bergsma et al, 2013 [38]	Multinomial (>4)	SVM	Name and name clusters	0.81	N/A	N/A
Nguyen et al, 2018 [66]	Multinomial (>4)	Neural network	Images	0.53	N/A	N/A

^a^ML: machine learning.

^b^GBDT: gradient-boosted decision tree.

^c^N/A: not applicable.

^d^SVM: support vector machine.

^e^DLLP: deep learning from label proportions.

^f^LR: logistic regression.

^g^CNN: convolutional neural network.

^h^NR: not reported.

^i^EN: elastic net.

### Manual Screening

A total of 12 studies used manual techniques to classify Twitter users into race or ethnicity categories [21,36,40,49-51,57,65, 87-90]. These studies generally combined qualitative interpretations of recent tweets, information in user bios making an affirmation of racial or ethnic identity, or photographs or images in the user timeline or profile.

In most cases, tweets were first identified by text matching based on terms of interest in the research topic, such as having a baby with a birth defect [50], commenting on a controversial topic [57,89], or using potentially gang- or drug-related language [40]. Researchers then identified the tweet authors and, in most cases, assigned race or ethnicity through hand coding based on profile and timeline content. Some studies coded primarily based on self-identifying statements of race used in a tweet or in users’ bios, such as people stating that they are a *Black American* [49,50,88,90] or hashtags [36] (such as #BlackScientist). Others coded exclusively based on the research team’s attribution of racial identity through the examination of profile photographs [21,57] or avatar [87]. Some authors coded primarily with self-declarations, with secondary indicators, such as profile pictures, language, usernames, or other content [40,51,65,88,89]. In most cases, it appears reasonable to infer that coding was performed by the study authors or members of their research teams, with the exception of those using the crowdsourcing marketplace, Amazon Mechanical Turk [21,90].

The agreement among coders was sometimes measured, but validity and accuracy measurements were not generally included. A study [65], however, documented 78% reliability for coding race compared with census demographics, with Black and White users being coded accurately 90% of the time and Hispanic or Asian users being accurately coded between 45% and 60% of the time. The high accuracy of Black users was based on the higher likelihood of Black users to self-identify.

### Census-Driven Prediction

Another approach to predict race or ethnicity is to use demographic information from the national census and census-like data and transfer it to the social media cohort. The US-based studies largely used census-based race and ethnicity categories: Asian and Pacific Islander, Black or African American, Latino or Hispanic, Native American, and White. A UK-based study included the categories British and Irish, West European, East European, Greek or Turkish, Southeast Asian, other Asian, African and Caribbean, Jewish, Chinese, and other minorities [83].

We identified 14 studies [39,48,52,54,60,63,70,71,74,77,83-85, 95] that used census geographic data, census surname classification, or a combination of both. A total of 6 studies incorporated geographic census data [39,52,63,74,83,84]. For example, Blodgett et al [39] created a simple probabilistic model to infer a user’s ethnicity by matching geotagged tweets with census block information. They averaged the demographic values of all tweets by the user and assumed this to be a rough proxy for the user’s demographics. Stewart [74] collected tweets tagged with geolocation information (longitude and latitude). The ZIP code of the user was derived from this geolocation information and matched with the demographic information found in the ZIP Code Tabulation Area defined by the Census Bureau. This information was used to find a correlation between ethnicity and African American vernacular English syntax [74].

Other studies have used the census-derived name classification system to determine race or ethnicity based on user names. We identified 12 studies that predicted user race or ethnicity using surnames [48,54,60,63,70,71,77,83-85,95,189]. Surnames were used to assign race or ethnicity using either a US census-based name classification system or, less commonly, an author in-house generated classification system. Of these 12 studies, 7 (58%) relied solely on the user’s last names [48,54,60,63,70,71,85]. Of those that reported validating the system, validation methods of this name-based system alone were not reported, but 4 (33%) of the 12 studies reported an accuracy between 71.8% and 81.25% [63,70,71,83]. Of note, a study reported vastly different accuracies in predicting whiteness versus blackness (94% predicting White users vs 33% predicting African American or Black users) [83]. The remaining 2 studies augmented name-based predictions with aggregate demographic data from the American Community Survey or equivalent surveys. For example, statistical and text mining methods have been used to extract surnames from Twitter profiles, combining this information with census block information based on geolocated tweets to assess the probability of the user’s race or ethnicity [60]. However, these studies did not report validation or accuracy.

### Ad Hoc ML or NLP

A total of 24 papers [33-35,37,38,46,47,61,64,66-68,72,76, 78-82,91-94,99] used ML or NLP to automatically classify users based on their race or ethnicity. ML and NLP methods were used to process the data made available by Twitter users, such as profile images, tweets, and location of residence. These studies almost invariably consisted of larger cohorts, with considerable variation in the specific methods used.

Supervised ML models (in which some annotated data were used to *train* the system) were used in 12 (50%) of the 24 studies. The models used include support vector machine [38,46,61], gradient-boosted decision trees [67,68], and regression models [33,34,37,76,96].

Semisupervised (where a large set of unannotated data is also used for training the system, in addition to annotated data) or fully unsupervised models using neural networks or regression were used for classification in 10 (42%) of the 24 studies [33,35,66,72,78,79,81,92-94].

A total of 2 studies used an ensemble of previously published race or ethnicity classifiers by processing the data through 4 extant models and using a majority rule approach to classify users based on the output of each classifier [80,91].

ML models use features or data inputs to predict desired outputs. Features derived from textual information in the user’s profile description, such as name or location, have been used in some studies [34,35,38,60,67,68,79,81,92,93]. Other studies included features related to images, including but not exclusively profile images [46,67,68,189], and facial features in those images [66]. Some studies have used linguistic features to classify a user’s race or ethnicity [37,38,46,47,61,67,68,72,76,78,81,92-94,96]. Specific linguistic features used in the models include n-grams [38,46,72,91-94], topic modeling [46,61,78], sentiment and emotion [76], and self-reports [67,68,81]. Information about a user’s followers or network of friends was included as a feature in some studies under the assumption that members of these networks have similar traits [34,37,46,47,91].

Labeled data sets are used to train and test supervised and semisupervised ML models and to validate the output of unsupervised learning methods. Some of the studies used previously created data sets that contained demographic information, such as the MORPH longitudinal face database of images [189], a database of mugshots [38], or manually annotated data from previous studies [79,81]. Others created ground truth data sets from surveys [96] or by semiautomatic means, such as matching Twitter users to voter registrations [37], using extracted self-identification from user profiles or tweets [67,68,81], or using celebrities with known ethnicities [66]. Manual annotation of Twitter users was also used based on profile metadata [34,35,46,76], self-declarations in the timeline [61,82], or user images [35,94]. Table 2 summarizes the best performing ML approach, features used, and the reported results for each study that used automatic classification methods. In the table, the classifier is the number of race or ethnicity classification groups, ML model is the top performing algorithm reported, and features are the variables used in the predictions.

Data from Twitter are inherently imbalanced in terms of race and ethnicity. In ML, it is important to attempt to mitigate the effects of the imbalance, as the models have difficulty learning from a few examples and will tend to classify to the majority class and ignore the minority class. Few studies (12/67, 18%) have directly addressed this imbalance. Some opted to make the task binary, focusing only on their group of interest versus all others [67,68,94] or only on the majority classes [38,76]. Others choose modified performance metrics that account for imbalance when reporting their results [33,61,82]. A group, which was classified based on images, supplemented their training set from an additional data source for the minority classes [33,35]. Only 2 studies have experimented with comparator models trained on balanced data sets. In a study by Wood-Doughty et al [81], the majority class was undersampled in their training sets and [96] the minority classes were oversampled. In both cases, the overall performance of the models decreased in accuracy from 0.83 to 0.41 (on their best performing unbalanced model) and 0.84 to 0.68. [96], as the performance boost from the models, the superior performance on the majority class was eradicated.

### Off-the-shelf Software

A total of 17 studies [12,32,41-45,53,55,56,58,59,62,69,73, 75,86] used off-the-shelf software packages to derive race or ethnicity. Moreover, 10 studies [32,44,45,53,55,56,58,62,69,75] used Face++ [199], 5 studies [12,41-43,73] used Demographics Pro [200], and 2 studies used Onomap [201] software to determine ethnicity [59,86]. Face++ is a validated ML face detection service that analyzes features with confidence levels for inferred race attributes. Specifically, it uses deep learning to identify whether profile pictures contain a single face and then the race of the face (limited to Asian, Black, and White) and does not infer ethnicity (eg, Hispanic) [199]. Demographics Pro estimates the demographic characteristics based on Twitter behavior or use using NLP, entity identification, image analyses, and network theory [200]. Onomap is a software tool used for classifying names [201]. A total of 3 studies that used Face++ used the same baseline data set [45,62,75], and one used a partial subset of the same data set [69].

In total, 2 studies that used Face++ [32,58] did not measure its performance. Another study [44] stated that Face++ could identify race with 99% confidence or higher for 9% of total users. In addition, 2 studies [53,55] used Face++ along with other methods. One of these studies used Face++ in conjunction with demographics, using a given name or full name from a database that contains US census data for demographics. This study simply measured the percentage of Twitter users for which race data could be extracted (46% college students and 92% role models) but did not measure the performance of Face++ [53]. Another study [55] built a classifier model on top of using Face++ and recorded an accuracy of 83.8% when compared with users who stated their nationality.

A total of 4 studies [45,62,69,75] (with the same data set in full or in part) used the average confidence level reported by Face++ for race which was 85.97 (SD 0.024%), 85.99 (SD 0.03%), 86.12 (SD 0.032%), respectively, with a CI of 95%. When one of these studies [45] carried out its own accuracy assessment, they found an accuracy score of 79% for race when compared with 100 manually annotated pictures. Huang et al [56] also carried out an accuracy assessment and found that Face++ achieved an averaged accuracy score of 88.4% for race when compared with 250 manually annotated pictures.

A total of 5 studies [12,41-43,73] used Demographics Pro, and although they reported on Demographics Pro success in general, they did not directly report any metrics of its success. The 2 studies using Onomap provided no validation of the software [59,86].

**Figure 2:**
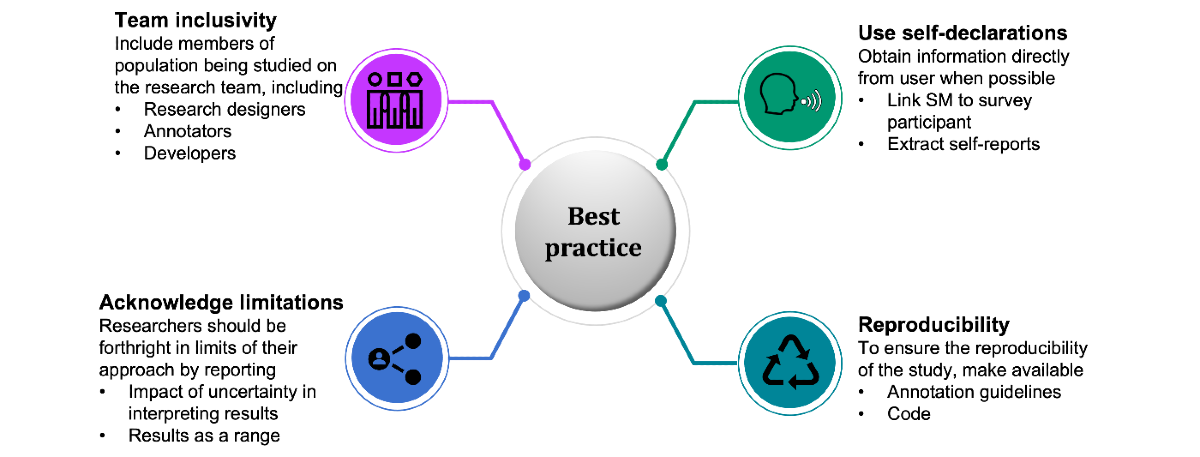
Summary of our best practice recommendations.

In light of our results, we have compiled our recommendations for best practice, which are summarized in Figure 2 and further examined in the Discussion section.

## Discussion

### Principal Findings

As there are no currently published guidelines or even best practice guidance, it is no surprise that researchers have used a variety of methods for estimating the race or ethnicity of Twitter users. We identified four categories for the methods used: manual screening, census-based prediction, ad hoc ML or NLP, and off-the-shelf software. All these methods exhibit particular strengths, as well as inherent biases and limitations.

Comparing the validity of methods for the purpose of deriving race or ethnicity is difficult as classification models differ not only in approach but also in the definition of the classification of race or ethnicity itself [112,202,203]. There is also a distinct lack of evaluation or validation of the methods used. Those that measured the performance of the methods used found accuracy to vary from 45% to 93%, with significantly lower accuracy in identifying categories of people of color.

This review sheds little light on the performance of commercial software packages. Previous empirical comparisons of facial recognition application programming interfaces have found that Face++ achieves 93% accuracy [204] and works comparatively better for men with lighter skins [205]. The studies included in our review suggested a lower accuracy. However, data on accuracy were not forthcoming in any of the included studies using Demographics Pro [200]. Even when performance is assessed, the methodology used may be biased if there are issues with the *gold standard* used to train the model.

In addition to the 4 overarching methods used, the studies varied in terms of the features used to determine or define race or ethnicity. Furthermore, the reliability of the features used to determine or define race or ethnicity for this purpose is questionable. Specifically, the use of Twitter users’ profile pictures, names, and locations, the use of unvalidated linguistic features attributed to racial groups (such as slang words, African American vernacular English, Spanglish, or Multicultural London English), and the use of training data that are prone to perpetuate biases (eg, police booking photos or mug shots) were all of particular concern.

### Issues Related to the Methods Used

Approaches that include or rely solely on profile pictures to determine race or ethnicity can introduce bias. First, not all users have a photograph as their profile picture, nor is it easy to determine whether the picture used is that of the user. A study on the feasibility of using Face++ found that only 30.8% of Twitter users had a detectable single face in their profile. A manual review of automatically detected faces determined that 80% could potentially be of the user (ie, not a celebrity) [206]. Human annotation may introduce additional bias, and studies have found systematic biases in the classification of people into racial or ethnic groups based on photographs [207,208]. Furthermore, humans tend to perceive their own race more readily than others [209,210]. Thus, race or ethnicity in the annotation team has an impact on the accuracy of their race or ethnicity labels, potentially skewing the sample labels toward the race or ethnicity of the annotators [211,212]. Given ML and NLP methods are trained on these data sets, the human biases transfer to automated methods, leading to poorly supervised ML and training, which has been shown to result in discrimination by the algorithm [213-215]. These concerns did not appear to be interrogated by the study designers. Without exception, they present categorization of persons into race or ethnicity, assuming that a subjective reading of facial features or idiomatic speech is the gold standard both for coding of race or ethnicity and for training and evaluation of automated methods.

Other methods, such as using geography or names as indicators of race, may also be unreliable. One could argue that the demographic profile for a geographic region is a better representation of race or ethnicity in the demographic environment than an individual’s race or ethnicity. Problems in using postcodes or locations to decipher individual social determinants are well documented [216]. The use of census data from an area that is too large may skew the results. Among the studies reviewed, some used census block data, which are granular, whereas others extrapolated from larger areas, such as city- or county-level data. For example, Saravanan [72] inferred the demographics of users in a city as a certain ethnic group based on a city with a large population of that group; however, no fine-grained analysis was performed either for the city chosen or for geolocation of the Twitter user. Thus, the validity of their assumption that a user in Los Angeles County is of Mexican descent [72] is questionable. As these data were then used to create a *race or ethnicity* dictionary of terms used by that group to train their model, the questionable assumption further taints downstream applications and results. The models also do not consider the differences between the demographics of Twitter users and the general demographics of the population.

In addition, census demographic data that uses names are also questionable because of name-taking in marriage and indiscernible names.

The practice of using a Twitter user’s self-reported race or ethnicity would provide a label with high confidence but restrict the amount of usable data and introduce a margin of error depending on the method used to extract such self-reports. For example, in a sample of 14 million users, >0.1% matched precise regular expressions created to detect self-reported race or ethnic identity [128]. Another study used mentions of keywords related to race or ethnicity in a user’s bio; however, limited validation was conducted to ensure that the mention was actually related to the user’s race or ethnicity [67,68]. This lack of information gathered from the profile information leads to sampling bias in the training of the models [152].

Some models trained on manually annotated data did not have high interannotator agreement; for example, Chen et al [46] crowdsourced annotation agreement measured at 0.45. This can be interpreted as weak agreement, with the percentage of reliable data being 15% to 35% [217]. Training a model on such weakly labeled data produces uncertain results.

It is not possible to assume the accuracy of black box proprietary tools and algorithms. The only race or ethnicity measure that seems empirically reliable is self-report, but this has considerable limitations. Thus, faulty methods continue to underpin digital health research, and researchers are likely to become increasingly dependent on them. The *gold standard* data required to know the demographic characteristics of the Twitter user is difficult to ascertain.

The methods that we highlight as best practices include directly asking the Twitter users. This can be achieved, for example, by asking respondents of a traditional survey for both their demographic data and their Twitter handles so that the data can be linked [96]. This was undertaken in the NatCen Social Research British Social Attitudes Survey 2015, which has the added benefit of allowing the study of the accuracy of further methods for deriving demographic data [20]. Contacting Twitter users may also provide a gold standard but is impractical, given the current terms of use of Twitter that might consider such contact a form of spamming [72,204,205,216]. A limitation of extracting race or ethnicity from social media is the necessity to oversimplify the complexity of racial identity. The categories were often limited to Black, White, Hispanic, or Asian. Note that *Hispanic* is considered *ethnicity* by the US census, but most studies in ML used it as a *race* category, more so than Asian (because of low numbers in this category). Multiple racial identities exist, particularly from an international perspective, which overlooks multiracial or primary and secondary identities. In addition, inferred identities may differ from self-identity, raising further issues.

Given the sensitive nature of the data, it is important as a *best practice* for the results of studies that derive race or ethnicity from Twitter data to be reproducible for validation and future use. The reproducibility of most of the studies in this review would be difficult or impossible, as only 5 studies were linked to available code or data [38,47,79,81,108]. Furthermore, there is limited information regarding the coding of the training data. None of the studies detailed their annotation schemas or made available annotation guidelines. Detailed guidelines as a *best practice* may allow recreation or extension of data sets in situations where the original data may not be shared or where there is data loss over time. This is particularly true of data collected from Twitter, where the terms of use require that shared data sets consist of only tweet IDs, not tweets, and that best efforts to delete IDs from the data set if the original tweet is removed or made private by the user be in place. Additional restrictions are placed on special use cases for sensitive information, prohibiting the storage of such sensitive information if detected or inferred from the user. Twitter explicitly states that information on racial or ethnic origin cannot be *derived or inferred* for an individual Twitter user and allows academic research studies to use only aggregate-level data for analysis [218]. It may be argued that this policy is more likely to be targeted at commercial activities.

### Strengths and Limitations

We did not limit our database searches and other methods by study design; however, we were unable to identify any previous reviews on the subject. To the best of our knowledge, this is the first review of methods used to extract race or ethnicity from social media. We identified studies from a range of disciplines and sources and categorized and summarized the methods used. However, we were unable to obtain information on the methodologies used by private-sector companies that created software for this purpose. Marketing and targeted advertising are common on social media and are likely to use race as a part of their algorithms to derive target users.

We did not limit our included papers to those in which the extraction of race or ethnicity was the primary focus. Although this can be conceived as a strength, it also meant that reporting of the methods used was often poor. The accurate recreation of the data lost was hampered by not knowing how decisions were made in the original studies, including what demographic definitions of race or ethnicity were used, or how accuracy was determined. This limited the assessment of the included studies. Few studies have validated the methods or conducted an error analysis to assess how often race is misapplied and those that did, rarely used the most appropriate gold standard. This makes it difficult to directly compare the results of the different approaches.

### Future Directions

Future studies should investigate their methodological approaches to estimate race or ethnicity, offering careful interpretations that acknowledge the significant limits of these approaches and their impact on the interpretation of the results. This may include reporting the results as a range that communicates the inherent uncertainty of the classification model. Social media data may best be used in combination with other information. In addition, we must always be mindful that race is a proxy measure for the much larger impact of being a particular race or ethnicity in a society. As a result, the variability associated with race and ethnicity might reveal more about the effects of racism and social stratification than about individual user attributes. To conduct this study ethically and rigorously, we recommend several practices that can help reduce bias and increase reproducibility.

We recommend acknowledging the researchers’ bias that can influence the conceptualization of the implementation of the study. Incorporating this reflexivity, as is common in qualitative research, allows for the identification of potential blind spots that weaken the research. One way to address homogenous research teams is through the inclusion of experts in race or ethnicity or in those communities being examined. These biases can also be reduced by including members of the study population in the research process as experts and advisers [219]. Although big data from social media can be collected without ever connecting with the people who contributed the data, it does not eliminate the ethical need for researchers to include representative perspectives in research processes. Examples of patient-engaged research and patient-centered outcomes research, community-based participatory research, and citizen science (public participation in scientific research) within the health and social sciences amply demonstrate the instrumental value and ethical obligation of intentional efforts to involve nonscientist partners in cocreation of research [219]. The quality of data science can be improved by seriously heeding the imperative, *Nothing about us without us* [219]. Documenting and establishing the diverse competence attributes of a research team should become a standard. Emphasizing the importance of diverse teams within the research process will contribute to social and racial justice in ways other than improving the reliability of research.

In terms of the retrieved data, the most reliable (though imperfect) method for ascertaining race was when users self-identified their racial affiliation. Further research on overcoming the limitations of availability and sample size may be warranted. Indeed, a hybrid model with automated methods and manual extraction may be preferred. For example, automation methods could be developed to identify potential self-declarations in a user profile or timeline, which can then be manually interpreted.

Finally, we call for greater reporting of the validation by our colleagues. Without error analysis, computational techniques would not be able to detect bias. Further research is needed to establish whether any bias is systematic or random, that is, whether inaccuracies favor one direction or another.

### Conclusions

We identified major concerns that affect the reliability of the methods and bias the results. There are also ethical concerns throughout the process, particularly regarding the inference of race or ethnicity, as opposed to the extraction of self-identity. However, the potential usefulness of social media research requires thoughtful consideration of the best ways to estimate demographic characteristics such as race and ethnicity [112]. This is particularly important, given the increased access to Twitter data [202,203].

Therefore, we propose several approaches to improve the extraction of race or ethnicity from social media, including representative research teams and a mixture of manual and computational methods, as well as future research on methods to reduce bias.

## Appendix

Multimedia Appendix 1 Search strategies and characteristics of included studies.

## Abbreviations

ML: machine learning
NIH: National Institutes of Health
NLP: natural language processing
PRISMA: Preferred Reporting Items for Systematic Reviews and Meta-Analyses

## Data Availability

The included studies are available on the web, and the extracted data are presented in Table S2 in Multimedia Appendix 1. A preprint of this paper is also available: Golder S, Stevens R, O’Connor K, James R, Gonzalez-Hernandez G. 2021. Who Is Tweeting? A Scoping Review of Methods to Establish Race and Ethnicity from Twitter Datasets. SocArXiv. February 14. doi:10.31235/osf.io/wru5q.

## References

[1] Golder S, Norman G, Loke YK (2015). Systematic review on the prevalence, frequency and comparative value of adverse events data in social media. Br J Clin Pharmacol.

[2] Sarker A, Ginn R, Nikfarjam A, O'Connor K, Smith K, Jayaraman S, Upadhaya T, Gonzalez G (2015). Utilizing social media data for pharmacovigilance: a review. J Biomed Inform.

[3] Bhattacharya M, Snyder S, Malin M, Truffa MM, Marinic S, Engelmann R, Raheja RR (2017). Using social media data in routine pharmacovigilance: a pilot study to identify safety signals and patient perspectives. Pharm Med.

[4] Convertino I, Ferraro S, Blandizzi C, Tuccori M (2018). The usefulness of listening social media for pharmacovigilance purposes: a systematic review. Expert Opin Drug Saf.

[5] Golder S, Smith K, O'Connor K, Gross R, Hennessy S, Gonzalez-Hernandez G (2021). A comparative view of reported adverse effects of statins in social media, regulatory data, drug information databases and systematic reviews. Drug Saf.

[6] Bychkov D, Young S (2018). Social media as a tool to monitor adherence to HIV antiretroviral therapy. J Clin Transl Res.

[7] Kalf RR, Makady A, Ten HR, Meijboom K, Goettsch WG, IMI-GetReal Workpackage 1 (2018). Use of social media in the assessment of relative effectiveness: explorative review with examples from oncology. JMIR Cancer.

[8] Golder S, O'Connor K, Hennessy S, Gross R, Gonzalez-Hernandez G (2020). Assessment of beliefs and attitudes about statins posted on Twitter: a qualitative study. JAMA Netw Open.

[9] Golder S, Bach M, O'Connor K, Gross R, Hennessy S, Gonzalez Hernandez G (2021). Public perspectives on anti-diabetic drugs: exploratory analysis of Twitter posts. JMIR Diabetes.

[10] Hswen Y, Naslund JA, Brownstein JS, Hawkins JB (2018). Monitoring online discussions about suicide among Twitter users with schizophrenia: exploratory study. JMIR Ment Health.

[11] Howie L, Hirsch B, Locklear T, Abernethy AP (2014). Assessing the value of patient-generated data to comparative effectiveness research. Health Aff (Millwood).

[12] Cavazos-Rehg PA, Krauss MJ, Costello SJ, Kaiser N, Cahn ES, Fitzsimmons-Craft EE, Wilfley DE (2019). "I just want to be skinny.": a content analysis of tweets expressing eating disorder symptoms. PLoS One.

[13] Ahmed W, Bath PA, Sbaffi L, Demartini G (2019). Novel insights into views towards H1N1 during the 2009 Pandemic: a thematic analysis of Twitter data. Health Info Libr J.

[14] Cook N, Mullins A, Gautam R, Medi S, Prince C, Tyagi N, Kommineni J (2019). Evaluating patient experiences in dry eye disease through social media listening research. Ophthalmol Ther.

[15] Roccetti M, Salomoni P, Prandi C, Marfia G, Mirri S (2017). On the interpretation of the effects of the Infliximab treatment on Crohn’s disease patients from Facebook posts: a human vs. machine comparison. Netw Model Anal Health Inform Bioinforma.

[16] Madden ML, Cortesi S, Gasser U, Duggan M, Smith A, Beaton M (2013). Teens, social media, and privacy. Pew Internet & American Life Project.

[17] Chou WS, Hunt YM, Beckjord EB, Moser RP, Hesse BW (2009). Social media use in the United States: implications for health communication. J Med Internet Res.

[18] Social media use in 2018. Pew Research Center.

[19] Bowleg L, Teti M, Malebranche DJ, Tschann JM (2013). "It's an Uphill Battle Everyday": intersectionality, low-income black heterosexual men, and implications for hiv prevention research and interventions. Psychol Men Masc.

[20] Sloan L (2016). Social Science 'Lite'? Deriving demographic proxies from Twitter. The SAGE Handbook of Social Media Research Methods.

[21] McCormick TH, Lee H, Cesare N, Shojaie A, Spiro ES (2017). Using Twitter for demographic and social science research: tools for data collection and processing. Sociol Methods Res.

[22] Smedley A, Smedley BD (2005). Race as biology is fiction, racism as a social problem is real: anthropological and historical perspectives on the social construction of race. Am Psychol.

[23] Yudell M, Roberts D, DeSalle R, Tishkoff S (2020). NIH must confront the use of race in science. Science.

[24] Davenport L (2020). The fluidity of racial classifications. Annu Rev Polit Sci.

[25] Resident population and net change. U.S. Census Bureau.

[26] Zuberi T (2001). Thicker Than Blood How Racial Statistics Lie.

[27] Hardeman RR, Karbeah J (2020). Examining racism in health services research: a disciplinary self‐critique. Health Serv Res.

[28] Jenkins W, Schoenbach V, Rowley D, Ford C (2019). 2. Overcoming the impact of racism on the health of communities: what we have learned and what we have not. Racism: Science & Tools for the Public Health Professional.

[29] Jones CP (2018). Toward the science and practice of anti-racism: launching a national campaign against racism. Ethn Dis.

[30] Tricco AC, Lillie E, Zarin W, O'Brien KK, Colquhoun H, Levac D, Moher D, Peters MD, Horsley T, Weeks L, Hempel S, Akl EA, Chang C, McGowan J, Stewart L, Hartling L, Aldcroft A, Wilson MG, Garritty C, Lewin S, Godfrey CM, Macdonald MT, Langlois EV, Soares-Weiser K, Moriarty J, Clifford T, Tunçalp O, Straus SE (2018). PRISMA Extension for Scoping Reviews (PRISMA-ScR): checklist and explanation. Ann Intern Med.

[31] Updated guidance for trusted systematic reviews: a new edition of the Cochrane Handbook for Systematic Reviews of Interventions. Cochrane Database of Systematic Reviews.

[32] An J, Weber I (2016). # greysanatomy vs # yankees: demographics and hashtag use on Twitter. arXiv.

[33] (2017). Lightly supervised machine learning for classifying online social data. ProQuest.

[34] Ardehaly E, Culotta A (2015). Inferring latent attributes of Twitter users with label regularization. Proceedings of the 2015 Conference of the North American Chapter of the Association for Computational Linguistics: Human Language Technologies.

[35] Ardehaly E, Culotta A (2017). Co-training for demographic classification using deep learning from label proportions. Proceedings of the IEEE International Conference on Data Mining Workshops, ICDMW.

[36] Auguste D, Polman J, Miller S A data science approach to STEM (science, technology, engineering and math) identity research for African American communities. ProQuest.

[37] Barbera P (2017). Less is more? How demographic sample weights can improve public opinion estimates based on Twitter data. Work Paper NYU.

[38] Bergsma S, Dredze M, Van Durme B, Wilson T, Yarowsky D (2013). Broadly improving user classification via communication-based name and location clustering on Twitter. Proceedings of the 2013 Conference of the North American Chapter of the Association for Computational Linguistics: Human Language Technologies.

[39] Blodgett S, Wei J, O'Connor B (2018). Twitter Universal Dependency Parsing for African-American and Mainstream American English. Proceedings of the 56th Annual Meeting of the Association for Computational Linguistics (Volume 1: Long Papers).

[40] Borradaile G, Burkhardt B, LeClerc A (2020). Whose tweets are surveilled for the police: an audit of a social-media monitoring tool via log files. Proceedings of the 2020 Conference on Fairness, Accountability, and Transparency.

[41] Cavazos-Rehg P, Krauss M, Grucza R, Bierut L (2014). Characterizing the followers and tweets of a marijuana-focused Twitter handle. J Med Internet Res.

[42] Cavazos-Rehg PA, Krauss M, Fisher SL, Salyer P, Grucza RA, Bierut LJ (2015). Twitter chatter about marijuana. J Adolesc Health.

[43] Cavazos-Rehg PA, Zewdie K, Krauss MJ, Sowles SJ (2018). "No high like a brownie high": a content analysis of edible marijuana Tweets. Am J Health Promot.

[44] United we tweet?: a quantitative analysis of racial differences in twitter use. ResearchWorks Archive.

[45] Chakraborty A, Messiaso J, Benevenutoo F, Ghosh S, Ganguly N, Gummadi K (2017). Who makes trends? Understanding demographic biases in crowdsourced recommendations. Proceedings of the 11th AAAI International Conference on Web and Social Media (ICWSM ).

[46] Chen X, Wang Y, Agichtein E, Wang F (2021). A comparative study of demographic attribute inference in twitter. Proc Int AAAI Conf Web Social Media.

[47] Culotta A, Ravi NK, Cutler J (2016). Predicting Twitter user demographics using distant supervision from website traffic data. J Artificial Intell Res.

[48] De Choudhury M (2011). Tie formation on Twitter: homophily and structure of egocentric networks. Proceedings of the 2011 IEEE Third International Conference on Privacy, Security, Risk and Trust and 2011 IEEE Third International Conference on Social Computing.

[49] Firmansyah F, Jones J (2019). Did the black panther movie make blacks blacker? Examining black racial identity on twitter before and after the black panther movie release. Lecture Notes Comput Sci.

[50] Golder S, Chiuve S, Weissenbacher D, Klein A, O'Connor K, Bland M, Malin M, Bhattacharya M, Scarazzini LJ, Gonzalez-Hernandez G (2019). Pharmacoepidemiologic evaluation of birth defects from health-related postings in social media during pregnancy. Drug Saf.

[51] González Y, Cutter S Leveraging geotagged social media to monitor spatial behavior during population movements triggered by hurricanes. Scholar Common.

[52] Haffner M (2018). A spatial analysis of non-English Twitter activity in Houston, TX. Transact GIS.

[53] He L, Murphy L, Luo J (2016). Using social media to promote STEM education: matching college students with role models. Machine Learning and Knowledge Discovery in Databases.

[54] Hswen Y, Hawkins JB, Sewalk K, Tuli G, Williams DR, Viswanath K, Subramanian SV, Brownstein JS (2020). Racial and ethnic disparities in patient experiences in the United States: 4-year content analysis of Twitter. J Med Internet Res.

[55] Huang W, Weber I, Vieweg S (2014). Inferring nationalities of Twitter users and studying inter-national linking. Proceedings of the 25th ACM conference on hypertext and social media.

[56] Huang X, Xing L, Dernoncourt F, Paul M (2020). Multilingual Twitter corpus and baselines for evaluating demographic bias in hate speech recognition. 12th Language Resources and Evaluation Conference, European Language Resources Association.

[57] Karlsen AS, Scott KD (2019). Making sense of Starbucks’ anti-bias training and the arrests of two African American men: a thematic analysis of Whites’ Facebook and Twitter comments. Discourse Context Media.

[58] Kteily NS, Rocklage MD, McClanahan K, Ho AK (2019). Political ideology shapes the amplification of the accomplishments of disadvantaged vs. advantaged group members. Proc Natl Acad Sci U S A.

[59] Longley PA, Adnan M (2015). Geo-temporal Twitter demographics. Int J Geographical Inf Sci.

[60] Luo F, Cao G, Mulligan K, Li X (2016). Explore spatiotemporal and demographic characteristics of human mobility via Twitter: a case study of Chicago. Applied Geography.

[61] Markson C Detecting user demographics in twitter to inform health trends in social media. New Jersey Institute of Technology.

[62] Messias J, Vikatos P, Benevenuto F (2017). White, man, and highly followed: gender and race inequalities in Twitter. IEEE/WIC/ACM International Conference on Web Intelligence (WI'17).

[63] Mislove A, Lehmann S, Ahn Y, Onnela J-P, Rosenquist J (2011). Understanding the Demographics of Twitter Users. Proceedings of the International AAAI Conference on Web and Social Media.

[64] Mohammady E (2014). Using county demographics to infer attributes of Twitter users. Proceedings of the ACL Joint Workshop on Social Dynamics and Personal Attributes in Social Media.

[65] Murthy D, Gross A, Pensavalle A (2015). Urban social media demographics: an exploration of Twitter use in major American cities. J Comput Mediat Commun.

[66] Nguyen V, Tran M, Luo J (2018). Are French really that different? Recognizing Europeans from faces using data-driven learning. Proceedings of the 2018 24th International Conference on Pattern Recognition (ICPR).

[67] Pennacchiotti M, Popescu A-M. (2011). Democrats, republicans and starbucks afficionados: user classification in twitter. Proceedings of the 17th ACM SIGKDD international conference on Knowledge discovery and data mining.

[68] Pennacchiotti M, Popescu AM (2011). A machine learning approach to twitter user classification. Proc International AAAI Conference Web Social Media.

[69] Reis J, Kwak H, An J, Messias J, Benevenuto F (2017). Demographics of news sharing in the U.S. Twittersphere. Proceedings of the 28th ACM Conference on Hypertext and Social Media.

[70] Sadah SA, Shahbazi M, Wiley MT, Hristidis V (2015). A study of the demographics of web-based health-related social media users. J Med Internet Res.

[71] Sadah SA, Shahbazi M, Wiley MT, Hristidis V (2016). Demographic-based content analysis of web-based health-related social media. J Med Internet Res.

[72] Saravanan M (2017). Determining Ethnicity of Immigrants using Twitter Data. Proceedings of the 4th Multidisciplinary International Social Networks Conference.

[73] Sowles SJ, Krauss MJ, Connolly S, Cavazos-Rehg PA (2016). A content analysis of vaping advertisements on Twitter, November 2014. Prev Chronic Dis.

[74] Stewart I (2014). Now we stronger than ever: African-American English syntax in twitter. Student Research Workshop at the 14th Conference of the European Chapter of the Association for Computational Linguistics.

[75] Vikatos P, Messias J, Manoel M, Benevenuto F (2017). Linguistic diversities of demographic groups in twitter. Proceedings of the 28th ACM Conference on Hypertext and Social Media.

[76] Volkova S, Backrach Y (2018). Inferring perceived demographics from user emotional tone and user-environment emotional contrast. 54th Annual Meeting of the Association for Computational Linguistics (Volume 1: Long Papers).

[77] Wang W, Chi G Who are you? Estimating demographics of twitter users. PAA.

[78] Wang Y, Li Y, Luo J (2016). Deciphering the 2016 U.S. Presidential Campaign in the Twitter sphere: a comparison of the Trumpists and Clintonists. Proceedings of the 10th International AAAI Conference on Web and Social Media.

[79] Wood-Doughty Z, Andrews N, Marvin R, Dredze M (2018). Predicting Twitter User Demographics from Names Alone. Proceedings of the Second Workshop on Computational Modeling of People's Opinions, Personality, and Emotions in Social Media, Association for Computational Linguistics.

[80] Wood-Doughty Z, Smith M, Broniatowski D, Dredze M (2017). How Does Twitter User Behavior Vary Across Demographic Groups?. Proceedings of the Second Workshop on Natural Language Processing and Computational Social Science, Association for Computational Linguistics.

[81] Wood-Doughty Z, Xu P, Liu X, Dredze M (2021). Using noisy self-reports to predict twitter user demographics. Proceedings of the Ninth International Workshop on Natural Language Processing for Social Media, Association for Computational Linguistics.

[82] Xu S, Markson C, Costello KL, Xing CY, Demissie K, Llanos AA (2016). Leveraging social media to promote public health knowledge: example of cancer awareness via Twitter. JMIR Public Health Surveill.

[83] Ye J, Han S, Hu Y, Coskun B, Liu M, Qin H (2017). Nationality classification using name embeddings. Proceedings of the 2017 ACM on Conference on Information and Knowledge Management.

[84] Yin J, Chi G, Hook J (2018). Evaluating the representativeness in the geographic distribution of twitter user population. Proceedings of the 12th Workshop on Geographic Information Retrieval.

[85] (2021). Automated analysis of user-generated content on the web. ProQuest.

[86] Adnan M, Longley PA, Khan SM (2014). Social dynamics of Twitter usage in London, Paris, and New York City. First Monday.

[87] Coleman LS (2021). “we’re a part of this city, too”: an examination of the politics of representation of D.C. Native via #dcnativesday. Social Media Soc.

[88] Saha K, Yousuf A, Hickman L, Gupta P, Tay L, De Choudhury M (2021). A social media study on demographic differences in perceived job satisfaction. Proc ACM Human Comput Interaction (HCI).

[89] Hong T, Wu J, Wijaya D, Xuan Z, Fetterman J (2021). JUUL the heartbreaker: Twitter analysis of cardiovascularhealth perceptions of vaping. Tobacco Induced Diseases.

[90] Jiang J, Vosoughi S (2020). Not judging a user by their cover: understanding harm in multi-modal processing within social media research. Proceedings of the 2nd International Workshop on Fairness, Accountability, Transparency and Ethics in Multimedia: ACM.

[91] Mueller A, Wood-Doughty Z, Amir S, Dredze M, Nobles AL (2020). Demographic representation and collective storytelling in the me too twitter hashtag activism movement. Proc ACM Human Comput Interaction.

[92] Aguirre C, Harrigian K, Dredze M (2021). Gender and racial fairness in depression research using social media. Proceedings of the 16th Conference of the European Chapter of the Association for Computational Linguistics.

[93] Aguirre C, Dredze M (2021). Qualitative analysis of depression models by demographics. Proceedings of the Seventh Workshop on Computational Linguistics and Clinical Psychology: Improving Access.

[94] Gunarathne P, Rui H, Seidmann A (2019). Racial Discrimination in Social Media Customer Service: Evidence from a Popular Microblogging Platform.

[95] Ye J, Skiena S (2019). The Secret Lives of Names? Name Embeddings from Social Media. Proceedings of the 25th ACM SIGKDD International Conference on Knowledge Discovery and Data Mining.

[96] Preotiuc-Pietro D, Ungar L (2018). User-level race and ethnicity predictors from twitter text. Proceedings of the The 27th International Conference on Computational Linguistics; Association for Computational Linguistics.

[97] Hinds J, Joinson AN (2018). What demographic attributes do our digital footprints reveal? A systematic review. PLoS One.

[98] Abubakar U, Bashir SA, Abdullahi MB, Adebayo OS (2019). Comparative study of various machine learning algorithms for tweet classification. J Comput Sci.

[99] Ardehaly E, Culotta A, Raghavan V, Aluru S, Karypis G, Miele L (2017). Mining the demographics of political sentiment from twitter using learning from label proportions. Proceedings of the IEEE International Conference on Data Mining Workshops, ICDMW.

[100] An J, Ciampaglia GL, Grinberg N, Joseph K, Mantzarlis A, Maus G, Menczer F, Proferes N, Welles BF (2017). Reports of the workshops held at the 2017 international AAAI conference on web and social media. AI Magazine.

[101] Anindya I (2020). Understanding and mitigating privacy risks raised by record linkage. The University of Texas at Dallas.

[102] Bardier C Detecting electronic cigarette user disparity behaviors: an infovelliance study on twitter. ProQuest.

[103] Basterra L, Worthington T, Rogol J, Brown D (2017). Socio-Temporal Trends in Urban Cultural Subpopulations through Social Media.

[104] Beretta V, Maccagnola D, Cribbin T, Messina E (2015). An interactive method for inferring demographic attributes in twitter. Proceedings of the 26th ACM Conference on Hypertext & Social Media.

[105] Bergsma S, Van Durme B (2013). Using conceptual class attributes to characterize social media users. Proceedings of the 51st Annual Meeting of the Association for Computational Linguistics (Volume 1: Long Papers).

[106] Bi B, Shokouhi M, Kosinski M, Graepel T (2013). Inferring the demographics of search user: social data meets search queries. Proceedings of the International World Wide Web Conference Committee (IW3C2).

[107] Blevins T, Kwiatkowski R, Macbeth J, McKeown K, Patton D, Rambow O (2016). Automatically processing tweets from gang-involved youth: towards detecting loss and aggression. COLING 2016, the 26th International Conference on Computational Linguistics: Technical Papers.

[108] Blodgett S, Wei J, O'Connor B (2017). A dataset and classifier for recognizing social media english. Proceedings of the 3rd Workshop on Noisy User-generated Text: Association for Computational Linguistics.

[109] Bokányi E, Kondor D, Dobos L, Sebők T, Stéger J, Csabai I, Vattay G (2016). Race, religion and the city: twitter word frequency patterns reveal dominant demographic dimensions in the United States. Palgrave Commun.

[110] Racial identities on social media: projecting racial identities on Facebook, Instagram, and Twitter. Minnesota State University.

[111] Burnap P, Colombo G, Amery R, Hodorog A, Scourfield J (2017). Multi-class machine classification of suicide-related communication on Twitter. Online Soc Netw Media.

[112] Cesare N, Grant C, Nguyen Q, Lee H, Nsoesie E (2017). How well can machine learning predict demographics of social media users?. arXiv.

[113] Chan MS, Winneg K, Hawkins L, Farhadloo M, Jamieson KH, Albarracín D (2018). Legacy and social media respectively influence risk perceptions and protective behaviors during emerging health threats: a multi-wave analysis of communications on Zika virus cases. Soc Sci Med.

[114] Chenworth M, Perrone J, Love JS, Greller HA, Sarker A, Chai PR (2020). Buprenorphine initiation in the emergency department: a thematic content analysis of a #firesidetox Tweetchat. J Med Toxicol.

[115] Cheong M, Lee V (2009). Integrating web-based intelligence retrieval and decision-making from the twitter trends knowledge base. Proceedings of the 2nd ACM workshop on Social web search and mining.

[116] Chi G, Giles L, Kifer D, Van Hook J, Yin J (2017). Predicting twitter user demographics as a first step in big data for population research: developing unsupervised, scalable methods using real-time, large-scale twitter data. Proceedings of the 2017 International Population Conference.

[117] Claude F, Konow R, Ladra S (2016). Fast compressed-based strategies for author profiling of social media texts. Proceedings of the 4th Spanish Conference on Information Retrieval.

[118] Compton R, Lee C, Lu T, De Silva L, Macy M (2013). Detecting future social unrest in unprocessed Twitter data: “emerging phenomena and big data”. Proceedings of the 2013 IEEE International Conference on Intelligence and Security Informatics.

[119] Augmenting household travel survey and travel behavior analysis using large-scale social media data and smartphone GPS data. ProQuest.

[120] Dai H, Hao J (2017). Mining social media data for opinion polarities about electronic cigarettes. Tob Control.

[121] Daughton AR, Paul MJ (2019). Identifying protective health behaviors on Twitter: observational study of travel advisories and Zika virus. J Med Internet Res.

[122] DeJohn AD, Schulz EE, Pearson AL, Lachmar EM, Wittenborn AK (2018). Identifying and understanding communities using twitter to connect about depression: cross-sectional study. JMIR Ment Health.

[123] Diaz F, Gamon M, Hofman JM, Kıcıman E, Rothschild D (2016). Online and social media data as an imperfect continuous panel survey. PLoS One.

[124] (2018). Using social media to evaluate public acceptance of infrastructure projects. Digital Repository at University of Maryland.

[125] Eisenstein J (2013). Phonological factors in social media writing. Proceedings of the Workshop on Language in Social Media (LASM 2013).

[126] A case study on Black Twitter's reactions to the framing of blacks in Dove's 2017 Facebook advertisement. Digital Communs @ University of South Florida.

[127] Filho R, Almeida J, Pappa G (2015). Twitter Population Sample Bias and its impact on predictive outcomes: a case study on elections. Proceedings of the 2015 IEEE/ACM International Conference on Advances in Social Networks Analysis and Mining 2015.

[128] Filippova K (2012). User demographics and language in an implicit social network. Proceedings of the Joint Conference on Empirical Methods in Natural Language Processing and Computational Natural Language Learning.

[129] A world of one-way and two-way streetsxploring the nuances of fan-athlete interaction on Twitter. Indiana University.

[130] Georgiou T, Abbadi A, Yan X (2017). Privacy cyborg: towards protecting the privacy of social media users. Proceedings of the 2017 IEEE 33rd International Conference on Data Engineering (ICDE).

[131] Ghazouani D, Lancieri L, Ounelli H, Jebari C (2019). Assessing socioeconomic status of Twitter users: a survey. Proceedings of the International Conference on Recent Advances in Natural Language Processing (RANLP ).

[132] Gibbons J, Malouf R, Spitzberg B, Martinez L, Appleyard B, Thompson C, Nara A, Tsou M (2019). Twitter-based measures of neighborhood sentiment as predictors of residential population health. PLoS One.

[133] Gilchrist-Herring N (2020). An analysis of attitudes towards transgender individuals utilizing social media usage, ethnicity, gender, age range, and level of education. ProQuest.

[134] Giorgi S, Yaden DB, Eichstaedt JC, Ashford RD, Buffone AEK, Schwartz HA, Ungar LH, Curtis B (2020). Cultural differences in Tweeting about drinking across the US. Int J Environ Res Public Health.

[135] Profiling social media users with selective self-disclosure behavior. Singapore Management University (Singapore).

[136] Towards secure and privacy-preserving online social networking services. University of California, Berkeley.

[137] Gundecha P, Ranganath S, Feng Z, Liu H (2013). A tool for collecting provenance data in social media. Proceedings of the 19th ACM SIGKDD international conference on Knowledge discovery and data mining.

[138] Guo G, Zhu F, Chen E, Liu Q, Wu L, Guan C (2016). From footprint to evidence: an exploratory study of mining social data for credit scoring. ACM Trans Web.

[139] Gupta H, Lam T, Pettigrew S, Tait RJ (2018). The association between exposure to social media alcohol marketing and youth alcohol use behaviors in India and Australia. BMC Public Health.

[140] Haffner M (2018). A place-based analysis of #BlackLivesMatter and counter-protest content on Twitter. GeoJournal.

[141] Ikeda K, Hattori G, Ono C, Asoh H, Higashino T (2013). Twitter user profiling based on text and community mining for market analysis. Knowledge Based Syst.

[142] Ireland ME, Chen Q, Schwartz HA, Ungar LH, Albarracin D (2016). Action Tweets linked to reduced county-level HIV prevalence in the United States: online messages and structural determinants. AIDS Behav.

[143] Jha D, Singh R (2019). SMARTS: the social media-based addiction recovery and intervention targeting server. Bioinformatics.

[144] Jimenez S, Dueñas G, Gelbukh A, Rodriguez-Diaz C, Mancera S (2018). Automatic detection of regional words for pan-hispanic spanish on twitter. Proceedings of the Ibero-American Conference on Artificial Intelligence.

[145] Jones T (2015). Toward a description of African American vernacular English dialect regions using "Black twitter''. American Speech.

[146] Jørgensen A, Hovy D, Søgaard A (2016). Learning a POS tagger for AAVE-like language. Proceedings of the 2016 conference of the North American chapter of the association for computational linguistics: human language technologies.

[147] Kang Y, Zeng X, Zhang Z, Wang Y, Fei T (2018). Who are happier? Spatio-temporal analysis of worldwide human emotion based on geo-crowdsourcing faces. Proceedings of the Ubiquitous Positioning Indoor Navigation and Location Based Service (UPINLBS).

[148] Kent JD, Capello HT (2013). Spatial patterns and demographic indicators of effective social media content during theHorsethief Canyon fire of 2012. Cartography Geographic Inf Sci.

[149] (2019). Debiasing 2016 Twitter election analysis via Multi-Level Regression and Poststratification (MRP). University of Illinois.

[150] Kostakos P, Pandya A, Kyriakouli O, Oussalah M (2018). Inferring demographic data of marginalized users in twitter with computer vision APIs. Proceedings of the 2018 European Intelligence and Security Informatics Conference (EISIC).

[151] Kotzé E, Senekal B (2018). Employing sentiment analysis for gauging perceptions of minorities in multicultural societies: an analysis of Twitter feeds on the Afrikaner community of Orania in South Africa. J Transdisciplinary Res Southern Africa.

[152] Kumar D, Ukkusuri SV (2020). Enhancing demographic coverage of hurricane evacuation behavior modeling using social media. J Comput Sci.

[153] Lachlan KA, Spence PR, Lin X (2014). Expressions of risk awareness and concern through Twitter: on the utility of using the medium as an indication of audience needs. Comput Human Behav.

[154] Lama Y, Chen T, Dredze M, Jamison A, Quinn SC, Broniatowski DA (2018). Discordance between human Papillomavirus Twitter images and disparities in human Papillomavirus risk and disease in the United States: mixed-methods analysis. J Med Internet Res.

[155] (2016). Virtual Homespace (Re)constructing the Body and Identity Through Social Media.

[156] Lee-Won RJ, White TN, Potocki B (2017). The Black catalyst to tweet: the role of discrimination experience, group identification, and racial agency in Black Americans’ instrumental use of Twitter. Inf Commun Soc.

[157] Li J, Ritter A, Hovy E (2014). Weakly supervised user profile extraction from twitter. Proceedings of the 52nd Annual Meeting of the Association for Computational Linguistics (Volume 1: Long Papers).

[158] Lienemann BA, Unger JB, Cruz TB, Chu K (2017). Methods for coding tobacco-related Twitter data: a systematic review. J Med Internet Res.

[159] Lin Y (2014). Assessing sentiment segregation in urban communities. Proceedings of the International Conference on Social Computing.

[160] (2012). As seen on Twitter: African-American rhetorical traditions gone viral. Michigan University.

[161] Human activity recognition: a data-driven approach. UC Irvine.

[162] Lwowski B, Rios A (2021). The risk of racial bias while tracking influenza-related content on social media using machine learning. J Am Med Inform Assoc.

[163] Magdy A, Ghanem T, Musleh M, Mokbel M (2016). Understanding language diversity in local twitter communities. Proceedings of the 27th ACM Conference on Hypertext and Social Media.

[164] Maheshwari T, Reganti A, Chakraborty T, Das A (2017). Socio-ethnic ingredients of social network communities. Proceedings of the Companion of the ACM Conference on Computer Supported Cooperative Work and Social Computing.

[165] Meng H, Kath S, Li D, Nguyen QC (2017). National substance use patterns on Twitter. PLoS One.

[166] Montasser OK (2017). Predicting Demographics of High-Resolution Geographies with Geotagged Tweets. Proceedings of the Thirty-First AAAI Conference on Artificial Intelligence AAAI Press.

[167] Nguyen TT, Adams N, Huang D, Glymour MM, Allen AM, Nguyen QC (2020). The association between state-level racial attitudes assessed from Twitter data and adverse birth outcomes: observational study. JMIR Public Health Surveill.

[168] Mulders D, Bodt C, Bjell J, Pentl A, Verleysen M, Montjoye Y (2017). Improving individual predictions using social networks associativity. Proceedings of the 12th International Workshop on Self-Organizing Maps and Learning Vector Quantization, Clustering and Data Visualization (WSOM).

[169] Nelson J, Quinn S, Swedberg B, Chu W, MacEachren A (2015). Geovisual analytics approach to exploring public political discourse on Twitter. Isprs Int J Geo Inf.

[170] Nguyen QC, Kath S, Meng H, Li D, Smith KR, VanDerslice JA, Wen M, Li F (2016). Leveraging geotagged Twitter data to examine neighborhood happiness, diet, and physical activity. Appl Geogr.

[171] Nguyen QC, Li D, Meng H, Kath S, Nsoesie E, Li F, Wen M (2016). Building a national neighborhood dataset from geotagged Twitter data for indicators of happiness, diet, and physical activity. JMIR Public Health Surveill.

[172] Novak AN, Johnson K, Pontes M (2016). LatinoTwitter: discourses of Latino civic engagement in social media. First Monday.

[173] Odlum M, Cho H, Broadwell P, Davis N, Patrao M, Schauer D, Bales ME, Alcantara C, Yoon S (2020). Application of topic modeling to Tweets as the foundation for health disparity research for COVID-19. Stud Health Technol Inform.

[174] Oktay H, Firat A, Ertem Z (2014). Demographic breakdown of Twitter users: an analysis based on names. Proceedings of the Academy of Science and Engineering (ASE).

[175] Orsolini L, Papanti GD, Francesconi G, Schifano F (2015). Mind navigators of chemicals' experimenters? A web-based description of e-psychonauts. Cyberpsychol Behav Soc Netw.

[176] Pick J, Sarkar A, Rosales J (2019). Social media use in American counties: geography and determinants. Isprs Int J Geo Inf.

[177] (2017). Developing computational approaches to investigate health inequalities. University of Washington.

[178] Priante A, Hiemstra D, Saeed A, van den Broek T, Ehrenhard M, Need A (2016). #WhoAmI in 160 characters? Classifying social identities based on twitter profile descriptions. Proceedings of the First Workshop on NLP and Computational Social Science.

[179] Riederer C, Zimmeck S, Phanord C, Chaintreau A, Bellovin S (2015). I don't have a photograph, but you can have my footprints: revealing the demographics of location data. Proceedings of the ACM on Conference on Online Social Networks.

[180] Roberts MJ, Perera M, Lawrentschuk N, Romanic D, Papa N, Bolton D (2015). Globalization of continuing professional development by journal clubs via microblogging: a systematic review. J Med Internet Res.

[181] Roy S, Ghosh P (2021). A comparative study on distancing, mask and vaccine adoption rates from global Twitter trends. Healthcare.

[182] Rummo PE, Cassidy O, Wells I, Coffino JA, Bragg MA (2020). Examining the relationship between youth-targeted food marketing expenditures and the demographics of social media followers. Int J Environ Res Public Health.

[183] Runge K (2017). "Social" science, spider goats and American science audiences: investigating the effects of interpersonal networks on perceptions of emerging technologies. ProQuest.

[184] Sijtsma B, Qvarfordt P, Chen F (2016). Tweetviz: visualizing tweets for business intelligence. Proceedings of the 39th International ACM SIGIR conference on Research and Development in Information Retrieval.

[185] Singh M, Singh A, Bansal D, Sofat S (2019). An analytical model for identifying suspected users on Twitter. Cybernetics Syst.

[186] Tomeny TS, Vargo CJ, El-Toukhy S (2017). Geographic and demographic correlates of autism-related anti-vaccine beliefs on Twitter, 2009-15. Soc Sci Med.

[187] Tulloch J (2019). An appraisal of health datasets to enhance the surveillance of Lyme disease in the United Kingdom. ProQuest.

[188] Vydiswaran VG, Romero DM, Zhao X, Yu D, Gomez-Lopez I, Lu JX, Iott BE, Baylin A, Jansen EC, Clarke P, Berrocal VJ, Goodspeed R, Veinot TC (2020). Uncovering the relationship between food-related discussion on Twitter and neighborhood characteristics. J Am Med Inform Assoc.

[189] Wang Y, Feng Y, Luo J, Zhang X (2016). Voting with feet: who are leaving Hillary Clinton and Donald Trump. Proceedings of the IEEE International Symposium on Multimedia (ISM).

[190] Weeg C, Schwartz HA, Hill S, Merchant RM, Arango C, Ungar L (2015). Using Twitter to measure public discussion of diseases: a case study. JMIR Public Health Surveill.

[191] Wright M, Adams T (2019). #KnowBetterDoBetter: an examination of Twitter impact on disaster literacy. ProQuest.

[192] Yazdavar AH, Mahdavinejad MS, Bajaj G, Romine W, Sheth A, Monadjemi AH, Thirunarayan K, Meddar JM, Myers A, Pathak J, Hitzler P (2020). Multimodal mental health analysis in social media. PLoS One.

[193] Ying Q, Chiu D, Venkatramanan S, Zhang X (2018). Profiling OSN users based on temporal posting patterns. Proceedings of The Web Conference.

[194] Yuan F, Li M, Zhai W, Qi B, Liu R (2020). Social media based demographics analysis for understanding disaster response disparity. Proceedings of the Construction Research Congress 2020: Computer Applications.

[195] Zhang Z, Bors G (2019). “Less is more” : mining useful features from Twitter user profiles for Twitter user classification in the public health domain. Online Inf Rev.

[196] Zhao P, Jia J, An Y, Liang J, Xie L, Luo J (2018). Analyzing and predicting emoji usages in social media. Proceedings of the The Web Conference 2018.

[197] Zhong Y, Yuan N, Zhong W, Zhang F, Xie X (2015). You are where you go: inferring demographic attributes from location check-ins. Proceedings of the Eighth ACM International Conference on Web Search and Data Mining.

[198] Jiang Y, Li Z, Ye X (2018). Understanding demographic and socioeconomic biases of geotagged Twitter users at the county level. Cartography Geographic Inf Sci.

[199] Face++ homepage. Face++.

[200] Powerful audience demographics. DemographicsPro.

[201] Onomap is changing. Onomap.

[202] Academic research: preparing for the academic research application: learn everything there is to know about applying for the academic research product track. Twitter Inc.

[203] Twitter grants academics full access to public data, but not for suspended accounts. Reuters.

[204] Jung S, An J, Kwak H, Salminen J, Jansen B (2018). Assessing the accuracy of four popular face recognition tools for inferring gender, age, and race. Proceedings of the Twelfth International AAAI Conference on Web and Social Media.

[205] Buolamwini J, Gebru T (2018). Gender shades: intersectional accuracy disparities in commercial gender classification. 1st Conference on Fairness, Accountability and Transparency.

[206] Jung S, An J, Kwak H, Salminen J, Jansen B.J. (2017). Inferring social media users? Demographics from profile pictures: a face++ analysis on twitter users. Proceedings of The 17th International Conference on Electronic Business.

[207] (2020). Jointly de-biasing face recognition and demographic attribute estimationiasing Face Recognition and Demographic Attribute Estimation. Computer Vision – ECCV 2020.

[208] Fu S, He H, Hou Z (2014). Learning race from face: a survey. IEEE Trans Pattern Anal Mach Intell.

[209] Goldinger SD, He Y, Papesh MH (2009). Deficits in cross-race face learning: insights from eye movements and pupillometry. J Experimental Psychol Learn Memory Cognition.

[210] Meissner CA, Brigham JC (2001). Thirty years of investigating the own-race bias in memory for faces: a meta-analytic review. Psychol Public Policy Law.

[211] Jofre A, Berardi V, Brennan K, Cornejo A, Bennett C, Harlan J (2020). Crowdsourcing image extraction and annotation: software development and case study. Digital Humanities Q.

[212] King RD, Johnson BD (2016). A punishing look: skin tone and afrocentric features in the halls of justice. Am J Sociol.

[213] Cavazos JG, Phillips PJ, Castillo CD, O'Toole AJ (2021). Accuracy comparison across face recognition algorithms: where are we on measuring race bias?. IEEE Trans Biom Behav Identity Sci.

[214] Buolamwini J, Gebru T (2018). Gender shades: intersectional accuracy disparities in commercial gender classification. Proceedings of the 1st Conference on Fairness, Accountability and Transparency.

[215] Torralba A, Efros A (2011). Unbiased look at dataset bias. Proceedings of CVPR 2011.

[216] Moscrop A, Ziebland S, Bloch G, Iraola JR (2020). If social determinants of health are so important, shouldn’t we ask patients about them?. BMJ.

[217] McHugh ML (2012). Interrater reliability: the kappa statistic. Biochem Med (Zagreb).

[218] Developer terms: more about restricted uses of the Twitter APIs. Developer Platform.

[219] Alsaied T, Allen KY, Anderson JB, Anixt JS, Brown DW, Cetta F, Cordina R, D’udekem Y, Didier M, Ginde S, Di Maria MV, Eversole M, Goldberg D, Goldstein BH, Hoffmann E, Kovacs AH, Lannon C, Lihn S, Lubert AM, Marino BS, Mullen E, Pickles D, Rathod RH, Rychik J, Tweddell JS, Wooton S, Wright G, Younoszai A, Glenn T, Wilmoth A, Schumacher K (2020). The Fontan outcomes network: first steps towards building a lifespan registry for individuals with Fontan circulation in the United States. Cardiol Young.

