# Supplementary material for: Methods to Establish Race or Ethnicity of Twitter Users: Scoping Review
Source: J Med Internet Res. 2022 Apr 29;24(4):e35788. doi: 10.2196/35788 (PMC9107046; doi:10.2196/35788)
Supplement: Multimedia Appendix 1 [file jmir_v24i4e35788_app1.docx]

| Database | Most recent Date Searched | Total Number of Records |
| --- | --- | --- |
| ACL Anthology | 24/05/2021 | 100* |
| ACM Digital Library | 15/05/2021 | 150 |
| Cinahl | 10/06/2021 | 200 |
| Conference Proceedings Citation Index – Science (CPCI-S) | 15/05/2021 | 84 |
| Conference Proceedings Citation Index – Social Science (CPCI-SS) | 15/05/2021 | 7 |
| Emerging Sources Citation Index (ESCI) | 15/05/2021 | 41 |
| IEEE | 10/06/2021 | 186 |
| Library and Information Science Abstracts (LISA)‎ | 15/05/2021 | 120 |
| LISTA | 15/05/2021 | 79 |
| Ovid MEDLINE | 15/05/2021 | 84 |
| OpenGrey | 15/05/2021 | 0 |
| Proquest Dissertations & Theses: UK & Ireland | 15/05/2021 | 195 |
| Ovid PsycInfo | 15/05/2021 | 72 |
| Science Citation Index (SCI) | 15/05/2021 | 56 |
| Social Science Citation Index (SSCI) | 15/05/2021 | 111 |
| Zetoc | 15/05/2021 | 50 (including duplicates) |
| Internet search engines | | |
| Google Scholar | 15/05/2021 | 200* |
| Handsearching of Journals | | |
| Journal of Medical Internet Research | 24/05/2021 | Browsed |
| Handsearching of Conference Proceedings | | |
| International AAAI Conference on Weblogs and Social Media (ICWSM) | 24/05/2021 | Browsed |
| ACL proceedings | 24/05/2021 | Browsed |

## Multimedia Appendix 1

## Table S1: Search Strategies

**ACM Digital Library**

Searched: 15/05/2021

Results: 150 hits

(Twitter OR tweet OR tweeting OR tweets OR retweet* OR tweep*) in title or abstract

(Race OR racial OR ethnology OR Ethnic* OR latino* OR latina* OR latinx OR hispanic* OR whites OR Blacks OR caucasian* OR african-american* OR nonwhite OR nonwhite OR "native American" OR Demographic* OR Minority-group* OR Ethnic-Group* OR "Continental Population Groups" OR "black american" OR "black americans" OR "racial affiliation" OR "racial identity" OR "racial identification") in title or abstract

**Cinahl Complete**

Searched: 10/06/2021

Results: 200 hits

(Twitter OR tweet OR tweeting OR tweets OR retweet* OR tweep*) AND (Race OR racial OR ethnology OR Ethnic* OR latino* OR latina* OR latinx OR hispanic* OR whites OR Blacks OR caucasian* OR african-american* OR nonwhite OR nonwhite OR "native American" OR Demographic* OR Minority-group* OR Ethnic-Group* OR "Continental Population Groups" OR "black american" OR "black americans" OR "racial affiliation" OR "racial identity" OR "racial identification")

**IEEE Xplore**

Searched: 10/06/2021

Results: 186 hits

(Twitter OR tweet OR tweeting OR tweets OR retweet OR retweets OR tweep OR tweeps OR tweepy) AND (Race OR racial OR ethnology OR Ethnic* OR latino* OR latina* OR latinx OR hispanic* OR whites OR Blacks OR caucasian* OR african-american* OR nonwhite OR nonwhite OR "native American" OR Demographic* OR Minority-group OR Minority-groups OR Ethnic-Group OR Ethnic-Groups OR "Continental Population Groups" OR "black american" OR "black americans" OR "racial affiliation" OR "racial identity" OR "racial identification")

**Conference Proceedings Citation Index – Science (CPCI-S), Conference Proceedings Citation Index – Social Science (CPCI-SS), Social Science Citation Index (SSCI) and Science Citation Index (SCI), Emerging Sources**

Searched: Last update search: 15/05/2021

### Results: CPCI-S: 164 hits, CPCI-S: 7 hits, ESCI: 41 hits, SSCI: 111 hits and SCI: 56 hits

TS=( " Apriori association mining algorithm " OR " AdaBoost " OR " AdaBoost Decision Tree " OR " AdaBoostM1 " OR " Artificial Neural Networks " OR " Author's Own " OR " Absorbing Random Walk " OR " Associative Classification " OR " Attribute Selected Classifier " OR " Bayesian " OR " Bagging " OR " Bagging Tree " OR " Belief Based Mixture Modelling " OR " Batch Classification " OR " Bilinear Elastic Net " OR " Best First Tree " OR " Bayesian Logisic Regression " OR " Bayesian Multinomial Loistic Regression " OR " Bayes Network " OR " Bernoulli Naïve Bayes " OR " Boosting " OR " Back Propagation " OR " Boosted Tree Regression " OR " Balanced Winnow 2 " OR " C4.5 " OR " Clauset's Algorithm " OR " Classification and Regression Tree " OR " Convolutional Neural Networks " OR " Class Prior " OR " Covering Rules " OR " Conditional Random Fields " OR " Decision Table " OR " Discriminant Analysis " OR " Deep Averaging Networks " OR " Deep Multi-modal Multi-task " OR " Discriminitive Multinomial Naïve Bayes " OR " Decision Stump " OR " Decision Tree " OR " Early Branching " OR " Elastic Net " OR " Elastic Net Regression " OR " Feed-forward Neural Network " OR " Factor Graph Model " OR " FPGrowth Algorithm " OR " Gradient Boosted Decision Trees " OR " Gaussian Mixture Model " OR " Gaussian Process " OR " Gaussian Radial Basis Function Neural Network " OR " Gaussian Super Vector " OR " Hidden Markov Models " OR " Higher Order Singular Value Decomposition " OR " Instance-based Learning " OR " J48 " OR " JRip " OR " K Nearest Neighbours " OR " Logistic " OR " LADTree " OR " Lasso Regression " OR " Late Branching " OR " Logic Boost " OR " Latent Dirichlet Allocation " OR " Linear Discriminant Analysis " OR " Linear Regression " OR " Linear SVC " OR " LLDA Classifier " OR " LambdaMART " OR " Logic Model Trees " OR " Logistic Linear Regression " OR " Logistic Regression " OR " Logistic Regression with L2 Regularization " OR " Latent Semantic Analysis " OR " Latent Semantic Indexing " OR " M5P Tree " OR " Modified Balanced Winnow Neural Network " OR " Multi-class Classifier " OR " Multi-class Neural Networks " OR " Maximum Entropy " OR " Multi-task Elastic Net Regression " OR " Maximum Likelihood Estimation " OR " Multinomial Logistic Regression " OR " Multilayer Perceptron " OR " Multinomial Naïve Bayes " OR " Max Sum Algorithm " OR " Multi-task Bilinear Model " OR " Multi-task Learning " OR " Naïve Bayes " OR " Naïve Bayes Decision Tree " OR " Naïve Bayes Multinomial " OR " Naïve Classifier " OR " Non-negative Matrix Factorization " OR " Non-negative Tensor Factorization " OR " OneR " OR " Perceptron " OR " PART " OR " Principle Component Analysis " OR " Parallel Ensemble Method " OR " Probabilistic Neural Network " OR " Poisson Regression " OR " Patient Rule Induction Method " OR " Partially Supervised Soft Label " OR " Quadratic Discriminant Analysis " OR " Regression " OR " Random Sample Consensus " OR " Rule Based " OR " Radial Basis Function Network " OR " Radial Basis Function Neural Network " OR " Restrited Boltzmann Machine " OR " Reaction Diffusion Algorithm " OR " Regularized Extreme Learning Machine " OR " RepTree " OR " Random Forest " OR " Recurrent Neural Networks " OR " Ridge Regression " OR " Random Subspace " OR " Random Tree " OR " Rotation Forest " OR " Simple Cart " OR " Stochastic Gradient Descent " OR " Skip Gram " OR " Simple Logistic " OR " Simple Logistic Regression " OR " Support Vector Machines trained using Platt's sequential minimal optimization algorithm (SMO) " OR " Support Vector Machine " OR " Support Vector Machine Regression " OR " Universal Background Model " OR " Winnow " OR " Weighted Label Regularization " OR " XGBoost " OR " Machine learning " OR " Artificial intelligence " OR " Language Processing " OR " Algorithm classifier " OR " Algorithm Classification " OR " Feature extraction " OR " Deep Learning " OR " bag of words " OR " text mining " OR " text classification " OR " natural language processing " OR " gated recurrent unit " OR " word2vec " OR " K Nearest Neighbor " OR " long short term memory " OR “Face++” OR “DemographicsPro” OR “Demographics Pro” OR “face recognition” OR “Microsoft Azure” OR “Microsoft Face API” OR “RapidMiner”) AND ALL=(Twitter OR tweet OR tweeting OR tweets OR retweet* OR tweep*) AND ALL=(Race OR racial OR ethnology OR Ethnic* OR latino* OR latina* OR latinx OR hispanic* OR whites OR Blacks OR caucasian* OR african-american* OR nonwhite OR nonwhite OR "native American" OR Demographic* OR Minority-group* OR Ethnic-Group* OR "Continental Population Groups" OR "black american" OR "black americans" OR "racial affiliation" OR "racial identity" OR "racial identification" )

**Google Scholar**

Searched: 15/10/2020 and 15/05/2021

Search: twitter race ethnicity demographic

Results: 332,000 and 363,000 results

Sifted first 100 records on each date

**Library and Information Science Abstracts (LISA)‎**

## Searched: 15/05/2021

Results: 120 hits

SU(( " Apriori association mining algorithm " OR " AdaBoost " OR " AdaBoost Decision Tree " OR " AdaBoostM1 " OR " Artificial Neural Networks " OR " Author's Own " OR " Absorbing Random Walk " OR " Associative Classification " OR " Attribute Selected Classifier " OR " Bayesian " OR " Bagging " OR " Bagging Tree " OR " Belief Based Mixture Modelling " OR " Batch Classification " OR " Bilinear Elastic Net " OR " Best First Tree " OR " Bayesian Logisic Regression " OR " Bayesian Multinomial Loistic Regression " OR " Bayes Network " OR " Bernoulli Naïve Bayes " OR " Boosting " OR " Back Propagation " OR " Boosted Tree Regression " OR " Balanced Winnow 2 " OR " C4.5 " OR " Clauset's Algorithm " OR " Classification and Regression Tree " OR " Convolutional Neural Networks " OR " Class Prior " OR " Covering Rules " OR " Conditional Random Fields " OR " Decision Table " OR " Discriminant Analysis " OR " Deep Averaging Networks " OR " Deep Multi-modal Multi-task " OR " Discriminitive Multinomial Naïve Bayes " OR " Decision Stump " OR " Decision Tree " OR " Early Branching " OR " Elastic Net " OR " Elastic Net Regression " OR " Feed-forward Neural Network " OR " Factor Graph Model " OR " FPGrowth Algorithm " OR " Gradient Boosted Decision Trees " OR " Gaussian Mixture Model " OR " Gaussian Process " OR " Gaussian Radial Basis Function Neural Network " OR " Gaussian Super Vector " OR " Hidden Markov Models " OR " Higher Order Singular Value Decomposition " OR " Instance-based Learning " OR " J48 " OR " JRip " OR " K Nearest Neighbours " OR " Logistic " OR " LADTree " OR " Lasso Regression " OR " Late Branching " OR " Logic Boost " OR " Latent Dirichlet Allocation " OR " Linear Discriminant Analysis " OR " Linear Regression " OR " Linear SVC " OR " LLDA Classifier " OR " LambdaMART " OR " Logic Model Trees " OR " Logistic Linear Regression " OR " Logistic Regression " OR " Logistic Regression with L2 Regularization " OR " Latent Semantic Analysis " OR " Latent Semantic Indexing " OR " M5P Tree " OR " Modified Balanced Winnow Neural Network " OR " Multi-class Classifier " OR " Multi-class Neural Networks " OR " Maximum Entropy " OR " Multi-task Elastic Net Regression " OR " Maximum Likelihood Estimation " OR " Multinomial Logistic Regression " OR " Multilayer Perceptron " OR " Multinomial Naïve Bayes " OR " Max Sum Algorithm " OR " Multi-task Bilinear Model " OR " Multi-task Learning " OR " Naïve Bayes " OR " Naïve Bayes Decision Tree " OR " Naïve Bayes Multinomial " OR " Naïve Classifier " OR " Non-negative Matrix Factorization " OR " Non-negative Tensor Factorization " OR " OneR " OR " Perceptron " OR " PART " OR " Principle Component Analysis " OR " Parallel Ensemble Method " OR " Probabilistic Neural Network " OR " Poisson Regression " OR " Patient Rule Induction Method " OR " Partially Supervised Soft Label " OR " Quadratic Discriminant Analysis " OR " Regression " OR " Random Sample Consensus " OR " Rule Based " OR " Radial Basis Function Network " OR " Radial Basis Function Neural Network " OR " Restrited Boltzmann Machine " OR " Reaction Diffusion Algorithm " OR " Regularized Extreme Learning Machine " OR " RepTree " OR " Random Forest " OR " Recurrent Neural Networks " OR " Ridge Regression " OR " Random Subspace " OR " Random Tree " OR " Rotation Forest " OR " Simple Cart " OR " Stochastic Gradient Descent " OR " Skip Gram " OR " Simple Logistic " OR " Simple Logistic Regression " OR " Support Vector Machines trained using Platt's sequential minimal optimization algorithm (SMO) " OR " Support Vector Machine " OR " Support Vector Machine Regression " OR " Universal Background Model " OR " Winnow " OR " Weighted Label Regularization " OR " XGBoost " OR " Machine learning " OR " Artificial intelligence " OR " Language Processing " OR " Algorithm classifier " OR " Algorithm Classification " OR " Feature extraction " OR " Deep Learning " OR " bag of words " OR " text mining " OR " text classification " OR " natural language processing " OR " gated recurrent unit " OR " word2vec " OR " K Nearest Neighbor " OR " long short term memory ") ) AND (Twitter OR tweet OR tweeting OR tweets OR retweet* OR tweep*) AND (Race OR racial OR ethnology OR Ethnic* OR latino* OR latina* OR latinx OR hispanic* OR whites OR Blacks OR caucasian* OR african-american* OR nonwhite OR nonwhite OR "native American" OR Demographic* OR Minority-group* OR Ethnic-Group* OR "Continental Population Groups" OR "black american" OR "black americans" OR "racial affiliation" OR "racial identity" OR "racial identification" )

**LISTA**

Searched: last update 15/05/2021

Results: 79 hits

(" Artificial Neural Networks " OR " Associative Classification " OR " Batch Classification " OR " Bayes Network " OR " Boosting " OR " Back Propagation " OR " Classification and Regression Tree " OR " Convolutional Neural Networks " OR " Class Prior " OR " Conditional Random Fields " OR " Decision Table " OR " Discriminant Analysis " OR " Decision Stump " OR " Decision Tree " OR " Early Branching " OR " Elastic Net " OR " Elastic Net Regression " OR " Feed-forward Neural Network " OR " Factor Graph Model " OR " Gaussian Mixture Model " OR " Gaussian Process " OR " Hidden Markov Models " OR " Higher Order Singular Value Decomposition " OR " Instance-based Learning " OR " J48 " OR " JRip " OR " K Nearest Neighbours " OR " Logistic " OR " Lasso Regression " OR " Late Branching " OR " Latent Dirichlet Allocation " OR " Linear Discriminant Analysis " OR " Linear Regression " OR " Logistic Linear Regression " OR " Logistic Regression " OR " Latent Semantic Analysis " OR " Latent Semantic Indexing " OR " M5P Tree " OR " Multi-class Classifier " OR " Multi-class Neural Networks " OR " Maximum Entropy " OR " Maximum Likelihood Estimation " OR " Multinomial Logistic Regression " OR " Multilayer Perceptron " OR " Multinomial Naïve Bayes " OR " Multi-task Learning " OR " Naïve Bayes " OR " Naïve Classifier " OR " Non-negative Matrix Factorization " OR " Non-negative Tensor Factorization " OR " OneR " OR " Perceptron " OR " PART " OR " Principle Component Analysis " OR " Probabilistic Neural Network " OR " Poisson Regression " OR " Patient Rule Induction Method " OR " Quadratic Discriminant Analysis " OR " Regression " OR " Random Sample Consensus " OR " Rule Based " OR " Radial Basis Function Network " OR " Radial Basis Function Neural Network " OR " Reaction Diffusion Algorithm " OR " Regularized Extreme Learning Machine " OR " RepTree " OR " Random Forest " OR " Recurrent Neural Networks " OR " Ridge Regression " OR " Random Subspace " OR " Random Tree " OR " Rotation Forest " OR " Skip Gram " OR " Simple Logistic Regression " OR " Support Vector Machine " OR " Support Vector Machine Regression " OR " Universal Background Model " OR " Winnow " OR " XGBoost " OR " Stochastic gradient descent " OR " Machine learning " OR " Artificial intelligence " OR " Language Processing " OR " Algorithm classifier " OR " Algorithm Classification " OR " Feature extraction " OR " Deep Learning " OR " bag of words " OR " text mining " OR " text classification " OR " natural language processing " OR " gated recurrent unit " OR " word2vec " OR " K Nearest Neighbor " OR " long short term memory " OR Determin* OR Identif* OR Infer* OR Ascertain OR Establish* OR Predict* OR Classify OR Classification OR “Language Processing” OR “Algorithm Classifier “OR “intrinsic bias” OR “facial recognition” OR DemographicsPro OR Demographics Pro OR face recognition OR Microsoft Azure OR Microsoft Face API OR RapidMiner)

(Twitter OR tweet OR tweeting OR tweets OR retweet* OR tweep*)

(Race OR racial OR ethnology OR Ethnic* OR latino* OR latina* OR latinx OR hispanic* OR whites OR Blacks OR caucasian* OR african-american* OR nonwhite OR nonwhite OR "native American" OR Demographic* OR Minority-group* OR Ethnic-Group* OR "Continental Population Groups" OR "black american" OR "black americans" OR "racial affiliation" OR "racial identity" OR "racial identification")

**OpenGrey**

Searched: last update 15/05/2021

Results: 0 hits

(Twitter OR tweet OR tweeting OR tweets OR retweet* OR tweep*) AND (Race OR racial OR ethnology OR Ethnic* OR latino* OR latina* OR latinx OR hispanic* OR whites OR Blacks OR caucasian* OR african-american* OR nonwhite OR nonwhite OR "native American" OR Demographic* OR Minority-group* OR Ethnic-Group*)

**Proquest Dissertations & Theses: UK & Ireland**

Searched: last update search: 15/5/2021

Results: 195 hits

(" Artificial Neural Networks " OR " Associative Classification " OR " Batch Classification " OR " Bayes Network " OR " Boosting " OR " Back Propagation " OR " Classification and Regression Tree " OR " Convolutional Neural Networks " OR " Class Prior " OR " Conditional Random Fields " OR " Decision Table " OR " Discriminant Analysis " OR " Decision Stump " OR " Decision Tree " OR " Early Branching " OR " Elastic Net " OR " Elastic Net Regression " OR " Feed-forward Neural Network " OR " Factor Graph Model " OR " Gaussian Mixture Model " OR " Gaussian Process " OR " Hidden Markov Models " OR " Higher Order Singular Value Decomposition " OR " Instance-based Learning " OR " J48 " OR " JRip " OR " K Nearest Neighbours " OR " Logistic " OR " Lasso Regression " OR " Late Branching " OR " Latent Dirichlet Allocation " OR " Linear Discriminant Analysis " OR " Linear Regression " OR " Logistic Linear Regression " OR " Logistic Regression " OR " Latent Semantic Analysis " OR " Latent Semantic Indexing " OR " M5P Tree " OR " Multi-class Classifier " OR " Multi-class Neural Networks " OR " Maximum Entropy " OR " Maximum Likelihood Estimation " OR " Multinomial Logistic Regression " OR " Multilayer Perceptron " OR " Multinomial Naïve Bayes " OR " Multi-task Learning " OR " Naïve Bayes " OR " Naïve Classifier " OR " Non-negative Matrix Factorization " OR " Non-negative Tensor Factorization " OR " OneR " OR " Perceptron " OR " PART " OR " Principle Component Analysis " OR " Probabilistic Neural Network " OR " Poisson Regression " OR " Patient Rule Induction Method " OR " Quadratic Discriminant Analysis " OR " Regression " OR " Random Sample Consensus " OR " Rule Based " OR " Radial Basis Function Network " OR " Radial Basis Function Neural Network " OR " Reaction Diffusion Algorithm " OR " Regularized Extreme Learning Machine " OR " RepTree " OR " Random Forest " OR " Recurrent Neural Networks " OR " Ridge Regression " OR " Random Subspace " OR " Random Tree " OR " Rotation Forest " OR " Skip Gram " OR " Simple Logistic Regression " OR " Support Vector Machine " OR " Support Vector Machine Regression " OR " Universal Background Model " OR " Winnow " OR " XGBoost " OR " Stochastic gradient descent " OR " Machine learning " OR " Artificial intelligence " OR " Language Processing " OR " Algorithm classifier " OR " Algorithm Classification " OR " Feature extraction " OR " Deep Learning " OR " bag of words " OR " text mining " OR " text classification " OR " natural language processing " OR " gated recurrent unit " OR " word2vec " OR " K Nearest Neighbor " OR " long short term memory " OR Determin* OR Identif* OR Infer* OR Ascertain OR Establish* OR Predict* OR Classify OR Classification OR “Language Processing” OR “Algorithm Classifier “OR “intrinsic bias” OR “facial recognition” OR DemographicsPro OR Demographics Pro OR face recognition OR Microsoft Azure OR Microsoft Face API OR RapidMiner)

AND

(Twitter OR tweet OR tweeting OR tweets OR retweet* OR tweep*)

AND

(Race OR racial OR ethnology OR Ethnic* OR latino* OR latina* OR latinx OR hispanic* OR whites OR Blacks OR caucasian* OR african-american* OR nonwhite OR nonwhite OR "native American" OR Demographic* OR Minority-group* OR Ethnic-Group* OR "Continental Population Groups" OR "black american" OR "black americans" OR "racial affiliation" OR "racial identity" OR "racial identification")

**PsycInfo <1987 to March Week 2 2021> and Ovid MEDLINE(R) and Epub Ahead of Print, In-Process, In-Data-Review & Other Non-Indexed Citations and Daily 1946 to May 14, 2021**

## Searched: Last update search 15/05/2021

Results: PsycINFO: 72 hits and MEDLINE: 84 hits
1     (" Apriori association mining algorithm " OR " AdaBoost " OR " AdaBoost Decision Tree " OR " AdaBoostM1 " OR " Artificial Neural Networks " OR " Author's Own " OR " Absorbing Random Walk " OR " Associative Classification " OR "
Attribute Selected Classifier " OR " Bayesian " OR " Bagging " OR " Bagging Tree " OR " Belief Based Mixture Modelling " or " Batch Classification " OR " Bilinear Elastic Net " OR " Best First Tree " OR " Bayesian Logisic Regression " OR " Bayesian Multinomial Loistic Regression " OR " Bayes Network " OR " Bernoulli Naïve Bayes " OR " Boosting " OR " Back Propagation " OR " Boosted Tree Regression " OR " Balanced Winnow 2 " OR " C4.5 " OR " Clauset's Algorithm " OR "Classification and Regression Tree " OR " Convolutional Neural Networks " OR " Class Prior " OR " Covering Rules " OR "Conditional Random Fields " OR " Decision Table " OR " Discriminant Analysis " OR " Deep Averaging Networks " OR " Deep Multi-modal Multi-task " OR " Discriminitive Multinomial Naïve Bayes " OR " Decision Stump " OR " Decision Tree " OR "Early Branching " OR " Elastic Net " OR " Elastic Net Regression " OR " Feed-forward Neural Network " OR " Factor Graph Model " OR " FPGrowth Algorithm " OR " Gradient Boosted Decision Trees " OR " Gaussian Mixture Model " OR " Gaussian Process " OR " Gaussian Radial Basis Function Neural Network " OR " Gaussian Super Vector " OR " Hidden Markov Models " OR " Higher Order Singular Value Decomposition " OR " Instance-based Learning " OR " J48 " OR " JRip " OR " K Nearest Neighbours " OR " Logistic " OR " LADTree " OR " Lasso Regression " OR " Late Branching " OR " Logic Boost " OR " Latent Dirichlet Allocation " OR " Linear Discriminant Analysis " OR " Linear Regression " OR " Linear SVC " OR " LLDA Classifier " OR " LambdaMART " OR " Logic Model Trees " OR " Logistic Linear Regression " OR " Logistic Regression " or " Logistic Regression with L2 Regularization " OR " Latent Semantic Analysis " OR " Latent Semantic Indexing " OR " M5P Tree " OR " Modified Balanced Winnow Neural Network " OR " Multi-class Classifier " OR " Multi-class Neural Networks " OR " Maximum Entropy " OR " Multi-task Elastic Net Regression " OR " Maximum Likelihood Estimation " OR " Multinomial Logistic Regression " OR " Multilayer Perceptron " OR " Multinomial Naïve Bayes " OR " Max Sum Algorithm " OR "Multi-task Bilinear Model " OR " Multi-task Learning " OR " Naïve Bayes " OR " Naïve Bayes Decision Tree " OR " Naïve Bayes Multinomial " OR " Naïve Classifier " OR " Non-negative Matrix Factorization " OR " Non-negative Tensor Factorization " OR " OneR " OR " Perceptron " OR " PART " OR " Principle Component Analysis " OR " Parallel Ensemble Method " OR " Probabilistic Neural Network " OR " Poisson Regression " OR " Patient Rule Induction Method " OR " Partially Supervised Soft Label " OR " Quadratic Discriminant Analysis " OR " Regression " OR " Random Sample Consensus" OR " Rule Based " OR " Radial Basis Function Network " OR " Radial Basis Function Neural Network " OR " Restrited Boltzmann Machine " OR " Reaction Diffusion Algorithm " OR " Regularized Extreme Learning Machine " OR " RepTree " OR "Random Forest " OR " Recurrent Neural Networks " OR " Ridge Regression " OR " Random Subspace " OR " Random Tree " OR "Rotation Forest " OR " Simple Cart " OR " Stochastic Gradient Descent " OR " Skip Gram " OR " Simple Logistic " OR "Simple Logistic Regression " OR " Support Vector Machines trained using Platt's sequential minimal optimization algorithm (SMO) " OR " Support Vector Machine " OR " Support Vector Machine Regression " OR " Universal Background Model " OR " Winnow " OR " Weighted Label Regularization " OR " XGBoost " OR " Machine learning " OR " Artificial intelligence
" OR " Language Processing " OR " Algorithm classifier " OR " Algorithm Classification " OR " Feature extraction " OR " Deep Learning " OR " bag of words " OR " text mining " OR " text classification " OR " natural language processing " or " gated recurrent unit " OR " word2vec " OR " K Nearest Neighbor " OR " long short term memory " OR DemographicsPro OR Demographics Pro OR face recognition OR Microsoft Azure OR Microsoft Face API OR RapidMiner).mp.
2     (Twitter OR tweet OR tweeting OR tweets OR retweet* OR tweep*).mp.
3     (Race OR racial OR ethnology OR Ethnic* OR latino* OR latina* OR latinx OR hispanic* OR whites OR Blacks OR caucasian* OR african-american* OR nonwhite OR nonwhite OR "native American" OR Demographic* OR Minority-group* OR Ethnic-Group* OR "Continental Population Groups" OR "black american" OR "black americans" OR "racial affiliation" OR "racial identity" OR "racial identification").mp.
4     1 and 2 and 3

**Zetoc**

Searched: 15/05/2021

Results: 50 hits (including duplicates)

Multiple searches of the title field were carried out:

Twitter AND Race (10 hits)

Twitter AND Ethnic* (3 hits)

Twitter AND Demographic* (18 hits)

Twitter AND Racial (6 hits)

Twitter AND Ethnology (0 hits)

Twitter AND latino* (2 hits)

Twitter AND Latina* (1 hit)

Twitter AND latinx (0 hits)

Twitter AND hispanic* (1 hit)

Twitter AND whites (2 hits)

Twitter AND Blacks (1 hit)

Twitter AND caucasian* (0 hits)

Twitter AND african * (0 hits)

Twitter AND nonwhite (0 hits)

Twitter AND native American (1 hit)

Twitter AND Minorities (0 hits)

Twitter AND Continental Population Groups (0 hits)

Twitter AND black american* (2 hits)

# Table S2: Characteristics of Included Studies

| **Study** | **Study Country** | **Race / Ethnicity Categories** | **Classification Models/Software Used** | **Features and predictors** | **Twitter Users (N)** | | **Tweets/ Images (N)** | | **Performance Measures (e.g. accuracy, recall, precision, F1-score)** | **Validation Set (how obtained)** | **Paper Type** | |
| --- | --- | --- | --- | --- | --- | --- | --- | --- | --- | --- | --- | --- |
| **AD HOC Machine Learning/ NLP Approaches** | | | | | | | | | | | |  |
| Aguirre 2021 [94] | U.S. implied | African American/Black, Asian, Hispanic/Latino, White | Methods and data from Aguirre 2021 [95] | Text (unigrams); user name; profile metadata | 3750 |  | | NR | | Training dataset created from self-reports and manual labels [83] | Conference paper | |
| Aguirre 2021 [95] | U.S. implied | African American/Black, Asian, Hispanic/Latino, White | Methods from Wood-Doughty 2021 [83] | Text (unigrams); user name; profile metadata | 3750 |  | | Accuracy: 82.3%[3] [83] | | Training dataset created from self-reports and manual labels [83] | Conference paper | |
| Ardehaly 2015 [36] | U.S. | African American/Black, Asian, Hispanic/Latino, White | mLogR | County, names, followers; tweet language | 2,700,000 | 18,000,000 | | Accuracy: 61.9-82.3 | | Manual annotation of 770 user profiles (profile, tweets, image) | Conference paper | |
| Ardehaly 2017 [35] | U.S. | African American/Black, Asian, Hispanic/Latino, White | Multiple approaches reviewed in [36] [37] [66] | Profile picture; tweet text; self-declarations; last names; U.S. census name classification | 3,500,000 | 23,700,000 | | Reported in [36] [37] [66] | | Reported in [36] [37] [66] | Thesis | |
| Ardehaly 2017 [37] | U.S. | African American/Black, Hispanic/Latino, White; Black and White | deep LLP; Xception (image classification) | Profile images, tweets, name and county | 10,500 | 2,100,000 | | Text classification F1 score 0.73 - 0.92. Image classification f1-score: 0.77-0.95  classification | | manual annotation of 320 photos | Conference paper | |
| Barbera 2017 [39] | U.S. | African American/Black, Asian/Other, Hispanic/Latino, White, | LogR | Tweets, emojis, followers (bag of words, bag of emojis, bag of followers) | 233,132 |  | | Accuracy range: 75.00-98.00, Precision: 0.74-0.90, Recall: 0.02-0.98 Accuracy overall: 68.9-80.5 | | manual annotation of 2,000 user name and profile pictures | working paper | |
| Bergsma 2013 [40] | International | Origin: Indian, Non-Indian, Ethnicity: 13 European languages e.g. German, Dutch, French, Swedish | SVM | First and last names | 168,000,000 |  | | Accuracy: 48.50-81.30 (Ethnicity), 80.40-84.60 (Race) | | Ethnicity: database European football players (13,483) Race: mugshots mined from mugshots.com (7,977) | Conference paper | |
| Chen 2015 [48] | U.S. implied | African American/Black, Asian, Hispanic/Latino, White | SVM; names heuristic | Profile data: names, profile Images, self-descriptions, social network data, tweets | 1495 |  | | Accuracy: 46.00-79.00, AUC: 00.55-00.73, Precision: 00.54-00.74, Recall: 00.46-00.79 F1-scores: 0.48-0.75 | | manual annotation of 2266 users (AMT) | Conference paper | |
| Culotta 2016 [49] | U.S. implied | African American/Black, Asian, Hispanic/Latino, White | Regression model | Text, Twitter accounts user follows | 1836 |  | | 77% | | NR | Journal article | |
| Gunarathne 2019 [96] | U.S. | African American/Black, White, Other | CNN with binary classification AA/Black or not | Tweets from user timelines |  | 130,023 | | F1 Scores: 88% Averaged across all; 88.9% AA/Black | | 8187 users annotated based on profile image (2182 AA/Black, 3839 white, 1507 other); 5000 users, balanced dataset, used in training/testing | Conference paper | |
| Markson 2017 [63] | U.S. | African American/Black, Asian, Hispanic/Latino, White | SVM | Tweets of self-identified users | 779,653 |  | | ACC: 53 - 89% | | 4400 self-identified users | Thesis | |
| Ardehaly 2014 [66] | U.S. | African American/Black, Asian, Hispanic/Latino, White | LogR; Names Heuristic; ridgeR | Profile, profile picture, last names and tweets | 770 |  | | Accuracy: 45.19 -82.21, F1-score 00.32-00.81, Precision 00.48-00.83 | | manual annotation of 770 profiles,tweets,image); 2000 users keyword verified | Conference paper | |
| Mueller 2021 [93] | U.S. | African American/Black, Asian, Hispanic/Latino, White | Ensemble: EthSelfReportNeural Demographer [3, 10] | Text (unigram); Twitter accounts user follows | 256,650 | 660,237 | | 20.0% P and 32.4% R for Asian users, 50.8% P and 81.3% R for AA/black users, 52.2% P and 19.4% R for Hispanic users, and 93.3% P and 87.3% Rl for white users | | Data from [18] | Conference paper | |
| Nguyen 2018 [68] | International | French, German, Spanish, Italian, Russian. Saudi Arabian, Turkish, Egyptian, Japanese, Cuban, Peruvian | pretrained models -VGG-Net 16 and ResNet50 - on ImageNet | Images | 353,826 |  | | Accuracy: 53.2 | | language detection based on followers’ names, profile descriptions using Google Language APIs and Twitter language settings. | Conference paper | |
| Pennacchiotti 2011 [70] | U.S. implied | African American/Black, Non-African-American | GDBT | Profile picture, prototypical Words, prototypical Hashtags, generic LDA, domain Specific LDA, sentiment Words | 10,338 |  | | F1-score = 0.57 - 0.66, Precision = 0.53-0.88, Recall = 0.09-0.67 | | 3,000 self-identified (regex) African American users, 3000 non-African American identified | Conference paper | |
| Pennacchiotti [69] | U.S. implied | African American/Black, Not African-Americans | Gradient Boosted decision tree(GBDT) | Profile, messaging (tweeting) behavior, linguistic content of messages and social network information. | 14,000,000 |  | | F1 score: 0.611 - 0.703 | | 3,000 self-identified (regex) African American users, 3000 non-African American identified | Conference paper | |
| Preotiuc-Pietro 2018 [199] | U.S. | African American/Black, Asian, Hispanic/Latino, White | Logistic regression with Elastic Net | Tweet text |  | 5,415,985 | | All vs 1 Classification: AUC: 0.884(AA), 0.781(Latino), 0,832 Asian, 0.825 | | Users self-identified through survey | Conference paper | |
| Saravanan 2017 [74] | U.S. | Chinese, Indian, Mexican (Hispanic) | CNN (tensorflow) | Tweets | 6329 | 1,276,000 | | Not measured | | Population demographics | Conference paper | |
| Volkova 2018 [78] | U.S., Canada | African American/Black, White | LR; linear log models | User content; tweet level emotion and sentiment | 123,513 |  | | ROC AUC: 0.75 - 0.97 | | Manual annotation of 5,000 profiles [20] | Conference paper | |
| Wang 2016 [80] | U.S. | African American/Black, Hispanic/Latino, White | CNN(caffe); SVM classifier | Profile images using OpenCV |  | 58,291 | | Mean Average Precision: 95.6% | | MORPH database (training and validation). 55,134 sample images labeled for gender, race and age | Conference paper | |
| Wood-Doughty 2017 [82] | U.S. | African American/Black, Asian, Hispanic/Latino, White | Demographer, demographer with RNN; followers list; content classifier | User names; followers list; text | 820,000 | 158,000,000 | | NR | | none | Conference paper | |
| Wood-Doughty 2018 [81] | U.S. implied | African American/Black, Hispanic/Latino, White | CNN; RNN | Username; screen name | 4269 |  | | Accuracy: 44.3-65.9(3 way), 58.7-73.6(2 way) F1-score: 23.3-46.8(3 way), 44.3-71.7 (2 way) | | 4269 users labeled in previous studies | Conference paper | |
| Wood-Doughty [3] [83] | U.S. | African American/Black, Asian, Hispanic/Latino, White | CNN; LR; BERT | Text; user name; profile metadata | 278,000 (across 3 datasets) |  | | Imbalanced: F1-score: 0.25-0.46; Acc: 25-83.2%; Balanced: F1-score: 0.10 - 0.51; acc: 25-52.6% | | training dataset created from self reports and manual labels | Conference paper | |
| Xu 2016 [84] | U.S. | African American/Black, Asian, Hispanic/Latino, White | SVM and LDA | Tweet timeline | 779,653 |  | | Accuracy = 76.07%. | | 4400 self-identified users | Journal article | |
| **MANUAL APPROACHES** | | | | | | | | | | | |  |
| Auguste 2019 [38] | U.S. implied | African American/Black | Manual Coding | Self-declaration hashtags (e.g. Black people in Stem, Blackengineers, BlackScientists) | NR | | 152 | | NR | NR | Conference, dissertation | |
| Borradaile 2020 [42] | U.S. | African American/Black, Asian, Hispanic/Latino, White, Other, Don’t know | Manual Coding | Self-identification, photos or language | 788 | | 2932 | | NR | NR | Conference paper | |
| Coleman 2021 [89] | U.S. | Black | Manual Coding | Avatar | NR | | 249 | | NR | None | Journal article | |
| Firmansyah 2019 [51] | U.S. implied | African American/Black | Manual Coding (Anova) | Self-declared identifiers in bio (i.e., African/Afro American, black, woke) | NR | | NR | | NR |  | Journal article | |
| Golder 2018 [52] | U.S., International | African American/Black, Asian, Hispanic/Latino, White | Manual Coding | Tweets over 10 month timeline or profile self-declaration | 429 | | NR | | NR | none | Journal article | |
| Gonzalez 2019 [53] | U.S. | African American/Black, Asian, Hispanic/Latino, White | Manual Coding | Profile pictures, usernames, full name, description, URLs, multimedia content and tweets uploaded by the users served to manually estimate | 1,760 | | NR | | NR | none | Thesis | |
| Hong, 2021 [91] | U.S. | White, non-white, unknown | Manual Coding | Profile names, profile descriptions | 1,884,442 | | 1989 | | Inter-coder reliability (Cohen’s Kappa) 0.8 | None | Journal article | |
| Jiang, 2020 [92] | U.S. | White, non-white | Manual Coding (Amazon Turk) | Profile content | NR | | 100 | | NR | None | Conference abstract | |
| Karlsen 2019 [59] | U.S. Implied | White | Manual Coding | Profile pictures | NR | | 85 | | NR | none | Journal article | |
| McCormick 2017 [21] | U.S. | African American/Black, Asian, Hispanic/Latino, White | Manual Coding (Amazon Turk) | Profile pictures | 1,000 | | NR | | % Agreement: 71 (3 agree); 20-25 (2 agree)  Cohen Kappa 0.77–0.80 | none | Journal article | |
| Murthy 2016 [67] | U.S. | African American/Black, Asian, Hispanic/Latino, White, Other, Unknown | Manual Coding | Profile data, up to 100 recent tweets and image postings. | 4,900 | | NR | | Reliability: 78.3% | compared to Census demographics | Journal article | |
| Saha 2021 [90] | U.S. | White, Black, Hispanic, API | Manual Coding | Census name attribution and manual coding where explicit self-report is available in profile information | 1,500,000 | | 855,508 | | % agreement between 2 raters: 91% | None | Conference abstract | |
| **CENSUS DRIVEN APPROACHES** | | | | | | | | | | | |  |
| Blodgett 2018[37] [41] | U.S. | African American/Black, Asian, Hispanic/Latino, White |  | Distantly supervised mapping between authors and demographics |  | | 59,200,000 | | NR | NR | Conference paper | |
| De Choudhury 2011 [50] | US implied | African American/Black, Asian, Hispanic/Latino, White, Asian/PI, American Indian/Alaskan Native, 2+ races |  | Last names; U.S. census name classification | 450,000 | | 29,500,000 | | NR | NR | Conference paper | |
| Haffner 2018 [54] | U.S. | White, Foreign Born | regression modeling | Tweet language, compared to census data (% foreign born) | 5673 | |  | | NR | NR | Journal article | |
| Hswen 2020 [56] | U.S. | African American/Black, Asian, Hispanic/Latino, White, Asian/Pacific Islander, American Indian/Alaska Native persons | NR | Last names; U.S. census name classification |  | | 392,215 | | NR | NR | Journal article | |
| Luo 2016 [62] | U.S. | African American/Black, Asian, Hispanic/Latino, White, | Baysian Improved Surname Geocoding (BISG)) | Last names; U.S. census data | 3,010 | |  | | NR | none | Journal article | |
| Mislove 2011 [65] | U.S. | African American/Black, Hispanic/Latino, White, Asian/Pacific Islander | Probability based on name matched in census | Last names; U.S. census data | 54,981,152 | |  | | matched 71.8% users | US census | Conference paper | |
| Rivas 2021 [87] | U.S. | Asian, Black, Latino, White | Used classifier from Mislove 2011 [42] | Last names; U.S. census data | 142,411 | |  | | NR | none | Dissertation | |
| Sadah 2015 [72] | US | African American/Black, Asian, Hispanic/Latino, White | NR | Last names; U.S. census name classification | 5,095,849 | |  | | classifier accuracy: 81.25% | 50 users classified in each group in Google corpus | Journal article | |
| Sadah 2016 [73] | US | Asian, Hispanic/Latino, White | Ethnicity classifier from Sadah 2015[44] | Last names; U.S. census name classification |  | | 11,637,888 | | classifier accuracy: 81.25% | 50 users classified in each group in Google corpus | Journal article | |
| Stewart 2014 [76] | U.S. | African American/Black, No, Hispanic/Latino | LogR | Tweet dialect; geolocation of tweets | 1,135,019 | | 200,000,000 | | The constructions’ demographics deviated only slightly from the overall demographics, though the variation reflected the expected trend of higher African-American population (avg. +0.859%) and lower Caucasian population (avg.0.974%).Correlation co-efficients were uninformative comparing demographics. | population demographics | Conference paper | |
| Wang 2017 [79] | US | NR | Probability based on census data; spatial analysis (ACS data) | First and last name, census data | 800,000 | | 600,000,000 | | Not measured | NR | Conference paper | |
| Ye 2019 [97] | US implied | African American/Black, Asian, Hispanic/Latino, White; 39 nationalities | Naïve Bayes  (NamePrism developed in Ye 2017[49]) | Name embeddings, followers list | 286 Million Tweets | | 89 million users, | | Not Reported | US Census bureau: 58,407 White, 2,519 Black, 4,521 API and 5,346 Hispanic names | Conference paper | |
| Ye 2017 [85] | International | African American/Black, Asian, Hispanic/Latino, White, Asian/Pacific Islander (API), American Indian/Alaskan Native (AIAN), Two or more races | NB | First and last name; census data | 74,000,000 | |  | | Race/Ethnicity: Average F1: 0.73 (white: 0.94, AA/black: 0.33, Asian: 0.83, Hispanic: 0.86)  Nationality: Weighted Average: 0.79 | Labeled names from ethnic distribution of census | Conference paper | |
| Yin 2018 [86] | U.S. | African American/Black, Hispanic/Latino, White | Matched lastname to US Census name classification | Last names; U.S. census name classification, Census demographics |  | |  | | 52% of race/ethnicity identified | NR | Conference paper | |
| **"OFF THE SHELF" SOFTWARE** | | | | | | | | | | | |  |
| Adnan 2014 [88] | London, Paris, New York City | English, Irish, Scottish, Italian, Welsh, Spanish, Pakistani, Indian, Portuguese, Turkish, German, Jewish, Chinese, Polish, French | Onomap | User name | London: 140,919 users Paris: 42,729 users New York City: 59,272 users.  Number of users where ethnic group found London 99,974 Paris 23,450 New York City | | London: 2,412,252 Paris: 740,188 New York City: 646,053 | | NR | None |  | |
| An 2016 [34] | U.S. | African American/Black, Asian, White | Face++ | Profile image, tweets | 767,300 | | NR | | NR | None | Conference paper | |
| Cavazos-Rehg 2014 [43] | U.S.(79%), U.K. (6%), Canada, South Africa, Netherlands, Mexico | African-American/Black, Hispanic/Latino, White | DemographicsPro | Network-based data signals (e.g. ties between individuals, accounts followed, twitter usage), language in tweets and bios | 759,407 | | NR | | A confidence of 95% or above is required by DemographicsPro | Varies from 10,000-200,000 verified users depending on demographic inferred (as reported by DemographerPro) | Journal article | |
| Cavazos-Rehg 2015 [44] | U.S., Canada | African-American/Black, Hispanic/Latino, White | DemographicsPro | Network-based data signals (e.g. ties between individuals, accounts followed, twitter usage), language in tweets and bios | 6620 | | NR | | A confidence of 95% or above is required by DemographicsPro | Varies from 10,000-200,000 verified users depending on demographic inferred (as reported by DemographerPro) | Journal article | |
| Cavazos-Rehg 2017 [45] | U.S. Implied | African-American/Black, Asian, Hispanic/Latino, White | DemographicsPro | Network-based data signals (e.g. ties between individuals, accounts followed, twitter usage), language in tweets and bios | 2607 | | NR | | A confidence of 95% or above is required by DemographicsPro | Varies from 10,000-200,000 verified users depending on demographic inferred (as reported by DemographerPro) | Journal article | |
| Cavazos-Rehg 2019 [12] | U.S. Implied | African-American/Black, Hispanic/Latino, White | DemographicsPro | Network-based data signals (e.g. ties between individuals, accounts followed, twitter usage), language in tweets and bios | 1323 | | NR | | A confidence of 95% or above is required by DemographicsPro | Varies from 10,000-200,000 verified users depending on demographic inferred (as reported by DemographerPro) | Journal article | |
| Cesare 2017 [46] | U.S. | African American/Black, White | Face++ | Profile picture data of egos & alters; mechanical turkers, | 2402 | | NR | | Only accepted users with 99% confidence | NR | Dissertation | |
| Chakraborty 2017 [47] | U.S. | African American/Black, Asian, White | Face++ | Profile picture | 1,670,863 | | NR | | 85.99 +/- 0.03 | Manual annotation of 100 random users (accuracy of 79% compared to automatic labels) | Conference paper | |
| He 2016 [55] | U.S. | African American/Black, Asian/PI, Hispanic/Latino, White | Face++ (based on image) and Demographics (based on name) | User’s given or full names, profile pictures | 19545 | | NR | | NR | None | Conference paper | |
| Huang 2020 [58] | International | African American/Black, Asian, Hispanic/Latino, White | Face++ | Profile picture | 73,163 | | NR | | Accuracy=.884 | Manual annotations of 250 | Conference paper | |
| Huang, 2014 [57] | Qatar | Indian Subcontinent, Non-Qatari Arab, Others, Qatari, Southeast Asia, Westerner | Gradient boosted tree, Face++ | Location features, time zone, language, hashtags, profile features, name, ethnicity, UTF-8 charset type, Tweet source, mentioned users | 35,780 users; 5572765 profiles when friends and followers of users collected | | NR | | Accuracy 83.80, F-score 00.76 (Indian Subcontinent), 00.77 (Non-Qatari Arab), 00.54 (Others), 00.91 F1 Qatari, 00.92 F1 Southeast Asia 0.20 UN | 467 self-declared and manually annotated profiles | Conference paper | |
| Kteily 2019 [60] | U.S. implied | African-American/Black, White | Face++ | Profile pictures | 160,639 | | NR | | NR | None | Journal article | |
| Longley 2016 [61] | U.K. | British and Irish, West European, East European, Greek, Turkish, South East Asian, Other Asian, African & Caribbean, Jewish, Chinese, Other minority | Onomap software | Given names and family names | 98,607 | | NR | | NR | None | Journal article | |
| Messias 2017 [64] | U.S. | African American/Black, Asian, White | Face++ | Profile picture | 1,670,863 | | NR | | 85.97±0.024% for race, with a confidence interval of95% | NR | Conference paper | |
| Reis 2017 [71] | U.S. | African American/Black, Asian, White | Face++ | Profile picture | 937,308 | | NR | | Accuracy = 86.12 ± 0.032% for race, with a confidence interval of 95% | NR | Conference paper | |
| Sowles 2016 [75] | U.S. Implied | African-American/Black, Asian, Hispanic/Latino, White | DemographicsPro | Network-based data signals (e.g. ties between individuals, accounts followed, twitter usage), language in tweets and bios | 68567 | | NR | | NR | None | Journal article | |
| Vikatos 2017 [77] | U.S. | African American/Black, Asian, White | Face++ | Profile picture | 1,670,863 | | NR | | Accuracy = 85.97 + or -024% for race, with a confidence interval of 95% | NR | Conference paper | |
